# Supplementary material for: Metabolomics as a Potential Chemotaxonomical Tool: Application on the Selected Euphorbia Species Growing Wild in Serbia
Source: Plants (Basel). 2023 Jan 6;12(2):262. doi: 10.3390/plants12020262 (PMC9864624; doi:10.3390/plants12020262)
Supplement: Supplementary file 1 [file plants-12-00262-s001.zip › plants-2074166-supplementary.pdf]

Supplementary material

Metabolomics as a Potential Chemotaxonomical Tool: Application on the Selected *Euphorbia* Species Growing Wild in Serbia

Ivana Sofrenić[1], Boban Anđelković[1]* Dejan Gođevac[2], Stefan Ivanović[2], Katarina Simić[2], Jovana Ljujić[1], Vele Tešević[1] and Slobodan Milosavljević [1,3]

[1] University of Belgrade – Faculty of Chemistry, Studentski trg 12-16, 11000 Belgrade, Serbia;

[2] Institute of Chemistry, Technology and Metallurgy, National Institute of the Republic of Serbia, University of Belgrade, Njegoševa 12, 11000 Belgrade, Serbia

[3] Serbian Academy of Sciences and Arts, Knez Mihajlova 35, 11000 Belgrade, Serbia;

* Correspondence: aboban@chem.bg.ac.rs; Tel.: +381644425473

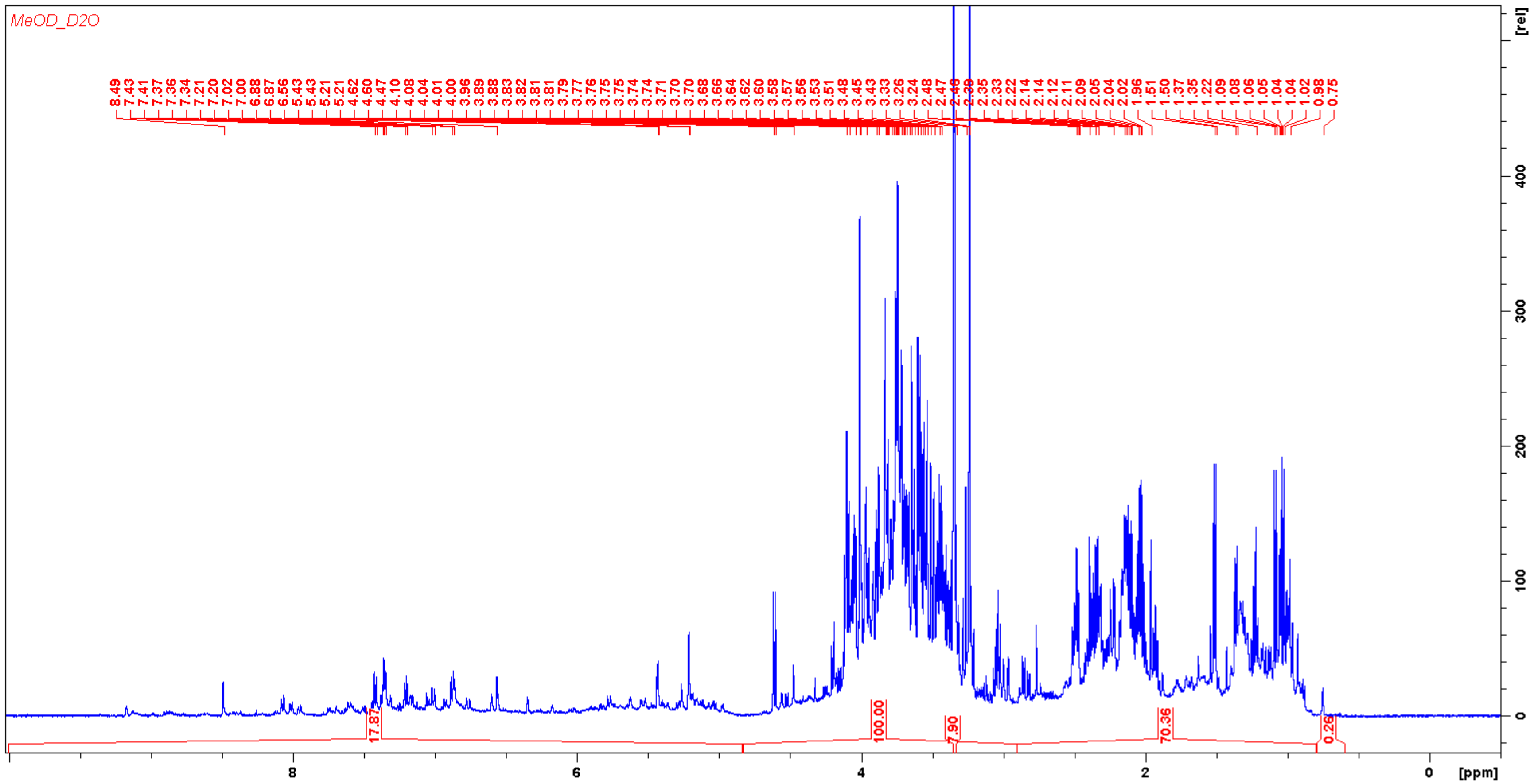

Figure S1: ^{1}H NMR spectrum of *E. salicifolia* extract of 1:1 mixture of deuterated methanol and potassium phosphate buffer in deuterated water.

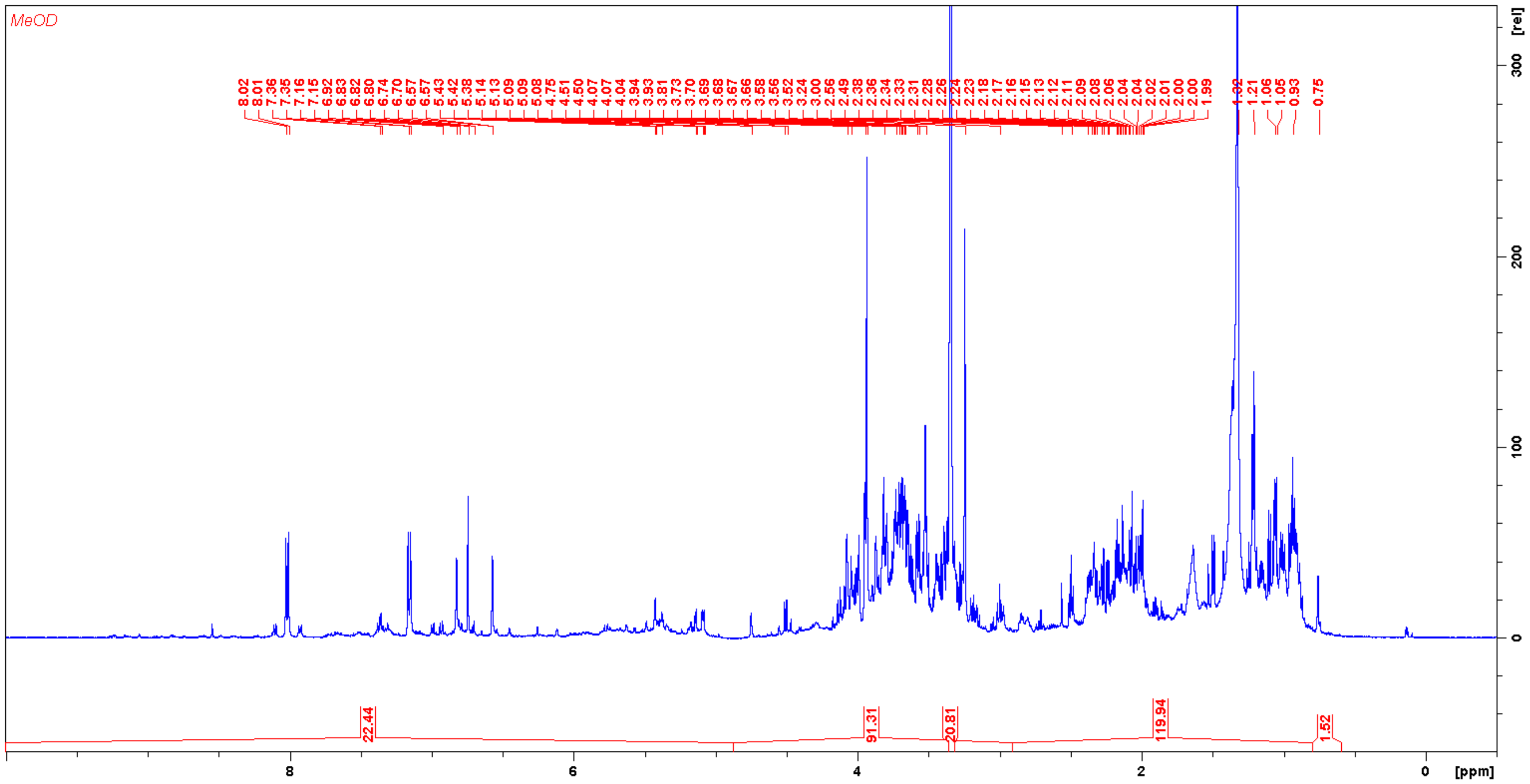

Figure S2: ^{1}H NMR spectrum of *E. salicifolia* extract of deuterated methanol.

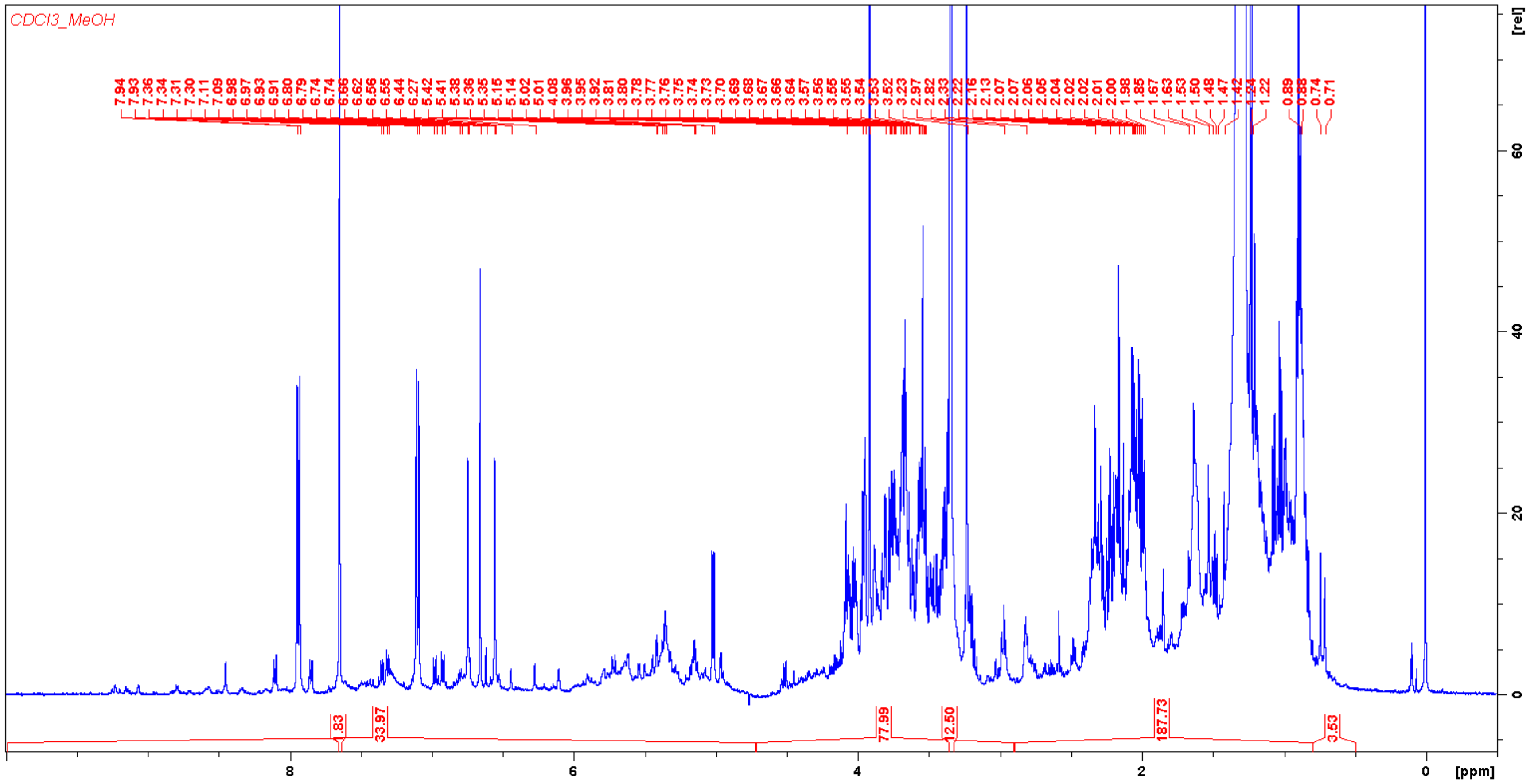

Figure S3: ^{1}H NMR spectrum of *E. salicifolia* extract of 1:1 mixture of deuterated methanol and deuterated chloroform.

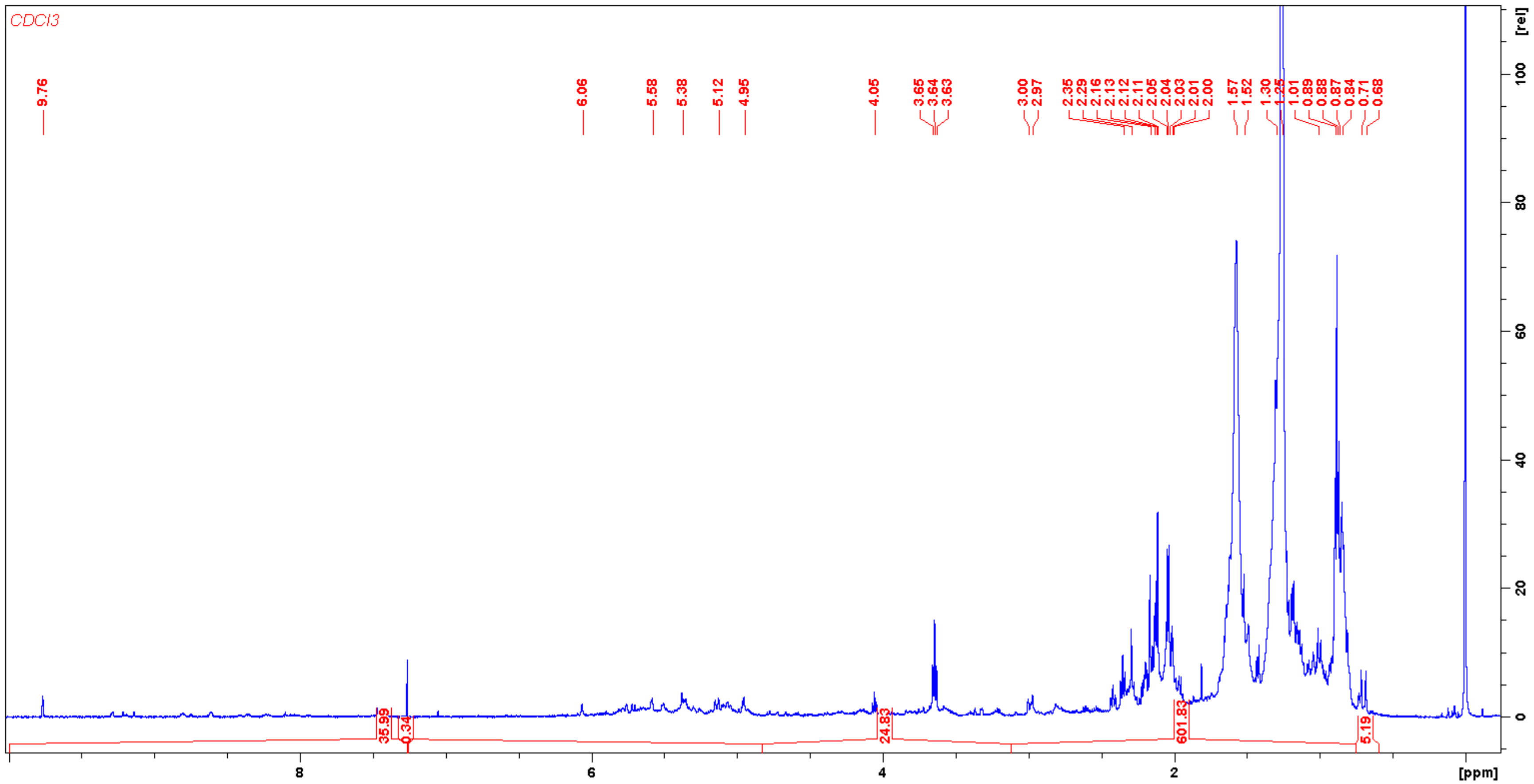

Figure S4: ^{1}H NMR spectrum of *E. salicifolia* extract of deuterated chloroform.

Table S1. Validation paremeters of CV-ANOVA test from OPLS-DA model of *E. segueriana vs*. remaining *Euphorbia* species.

M11	SS	DF	MS	F	p	SD
Total corr.	59	59	1			1
Regression	58.26919937	8	7.283649921	508.3110046	0	2.698820114
Residual	0.730785012	51	0.0143291			0.119704001

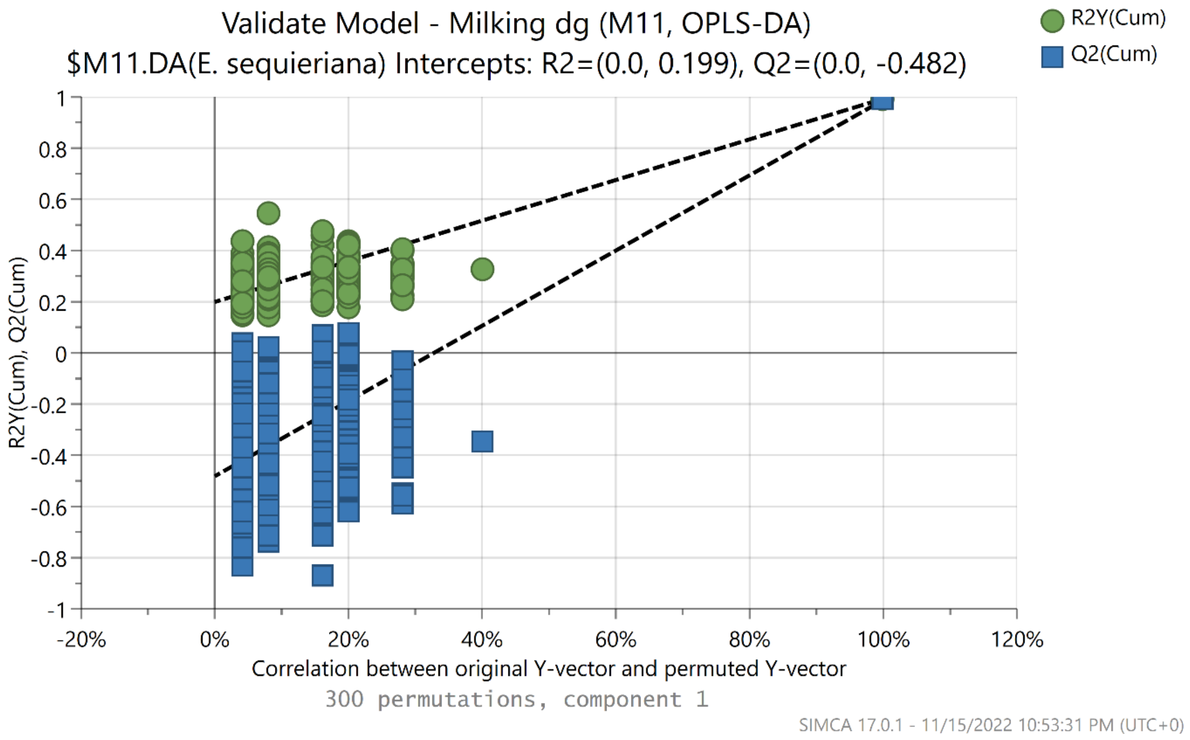

Figure S5: Permutation test of OPLS-DA model of *E. segueriana vs*. remaining *Euphorbia* species.

Table S2. Validation paremeters of CV-ANOVA test from OPLS-DA model of *E. salicifolia vs*. remaining *Euphorbia* species.

M12	SS	DF	MS	F	p	SD
Total corr.	59	59	1			1
Regression	58.50939941	8	7.313670158	760.2769775	0	2.704380035
Residual	0.490606993	51	0.00961975			0.0980803

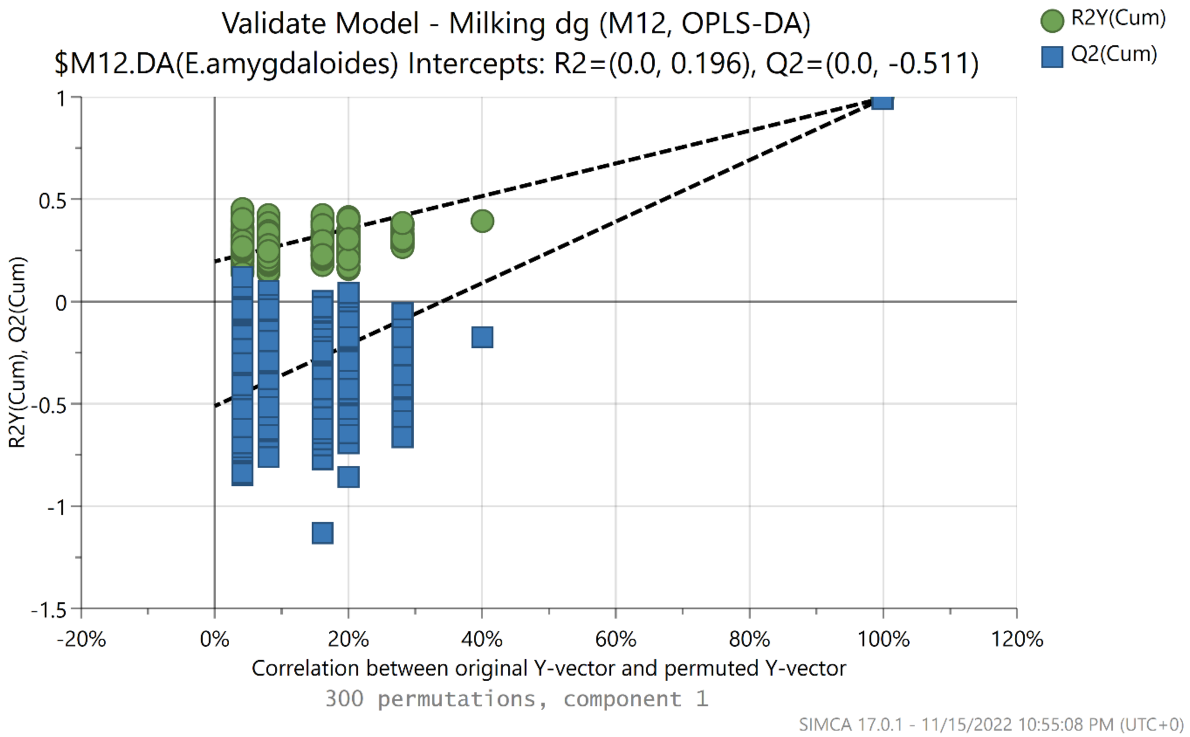

Figure S6: Permutation test of OPLS-DA model of *E. salicifolia vs*. remaining *Euphorbia* species.

Table S3. Validation paremeters of CV-ANOVA test from OPLS-DA model of *E. amygdaloides vs*. remaining *Euphorbia* species.

M13	SS	DF	MS	F	p	SD
Total corr.	59	59	1			1
Regression	57.87189865	8	7.233990192	327.0490112	0	2.689610004
Residual	1.128069997	51	0.022119001			0.148725003

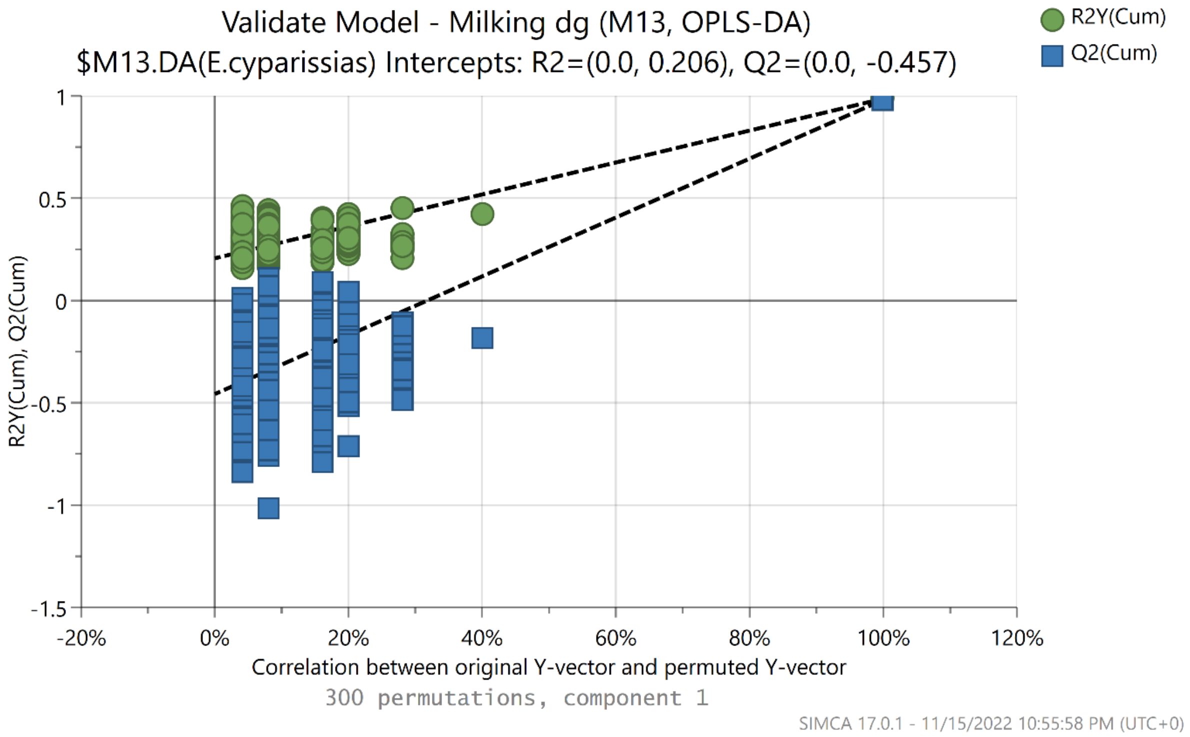

Figure S7: Permutation test of OPLS-DA model of *E. amygdaloides vs*. remaining *Euphorbia* species.

Table S4. Validation parameters of CV-ANOVA test from OPLS-DA model of *E. panonnica vs*. remaining *Euphorbia* species.

M14	SS	DF	MS	F	p	SD
Total corr.	59	59	1			1
Regression	57.73199844	8	7.216499805	290.2539978	0	2.686350107
Residual	1.268000007	51	0.024862699			0.157679006

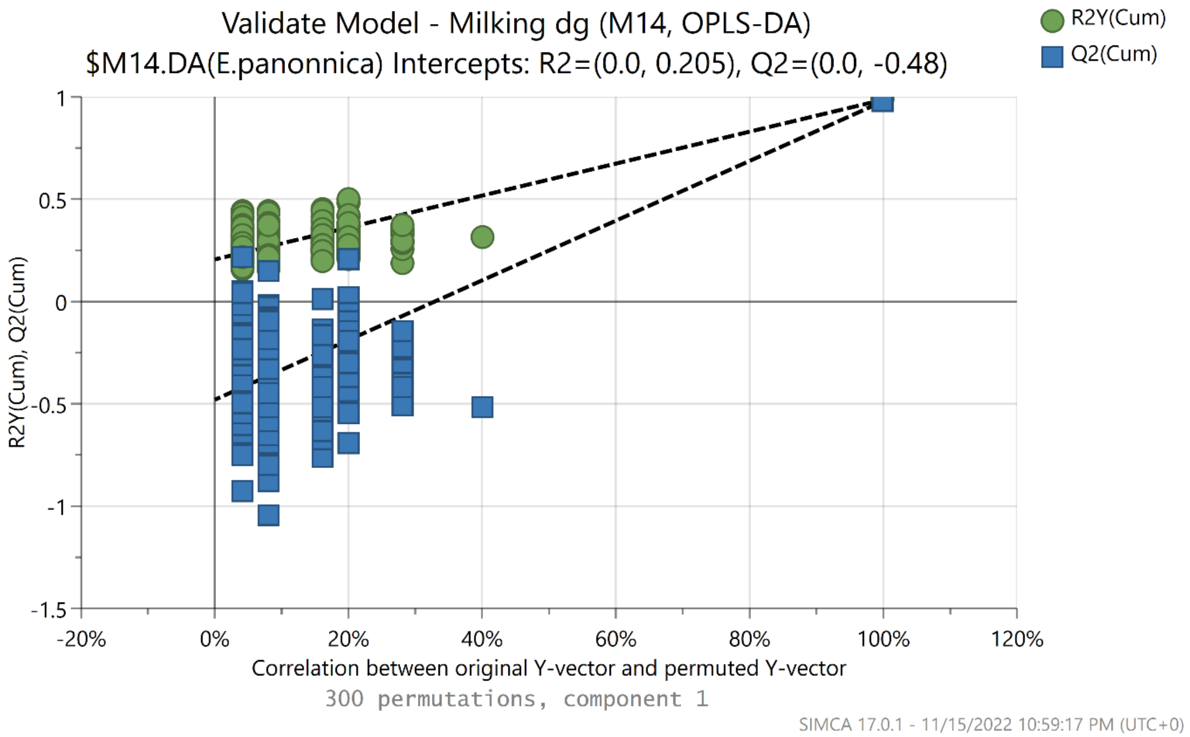

Figure S8: Permutation test of OPLS-DA model of *E. panonnica vs*. remaining *Euphorbia* species.

Table S5. Validation parameters of CV-ANOVA test from OPLS-DA model of *E. cyparissias vs*. remaining *Euphorbia* species.

M15	SS	DF	MS	F	p	SD
Total corr.	59	59	1			1
Regression	58.48870087	6	9.748109818	1010.400024	0	3.122200012
Residual	0.511334002	53	0.00964781			0.098223299

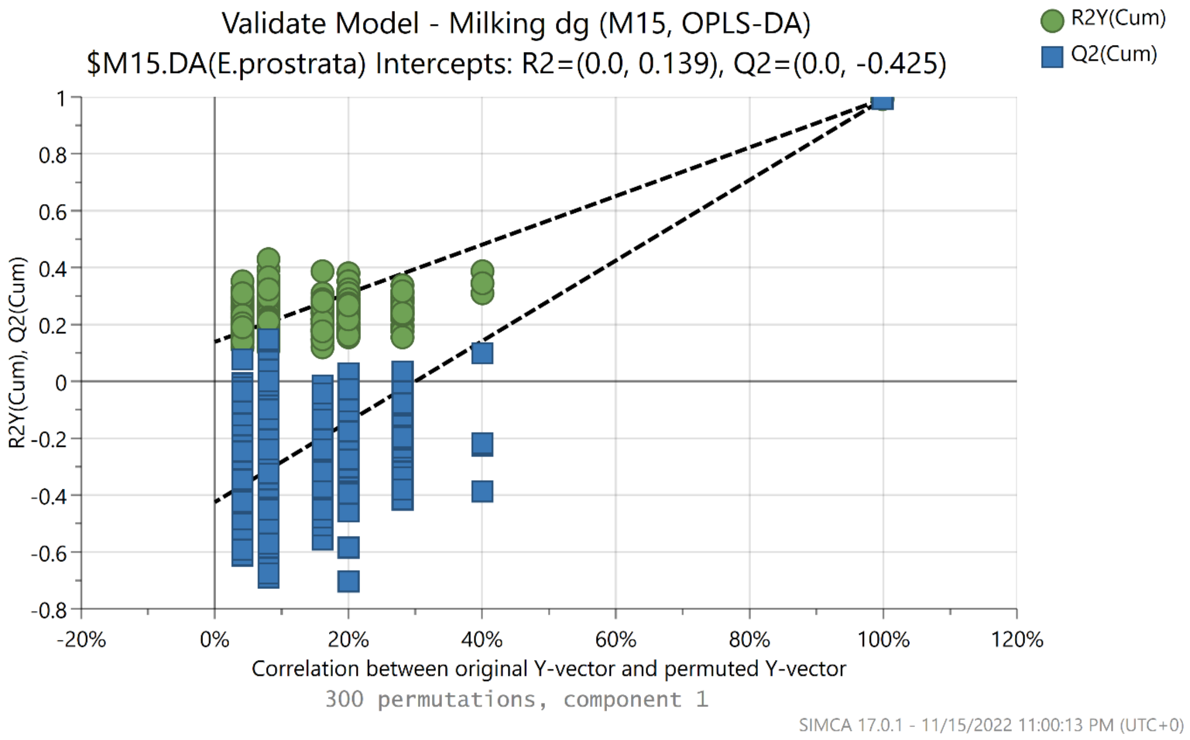

Figure S9: Permutation test of OPLS-DA model of *E. cyparissias vs*. remaining *Euphorbia* species.

Table S6. Validation parameters of CV-ANOVA test from OPLS-DA model of *E. maculata vs*. remaining *Euphorbia* species.

M16	SS	DF	MS	F	p	SD
Total corr.	59	59	1			1
Regression	58.3791008	4	14.5948	1292.719971	0	3.820310116
Residual	0.62094599	55	0.0112899			0.106253996

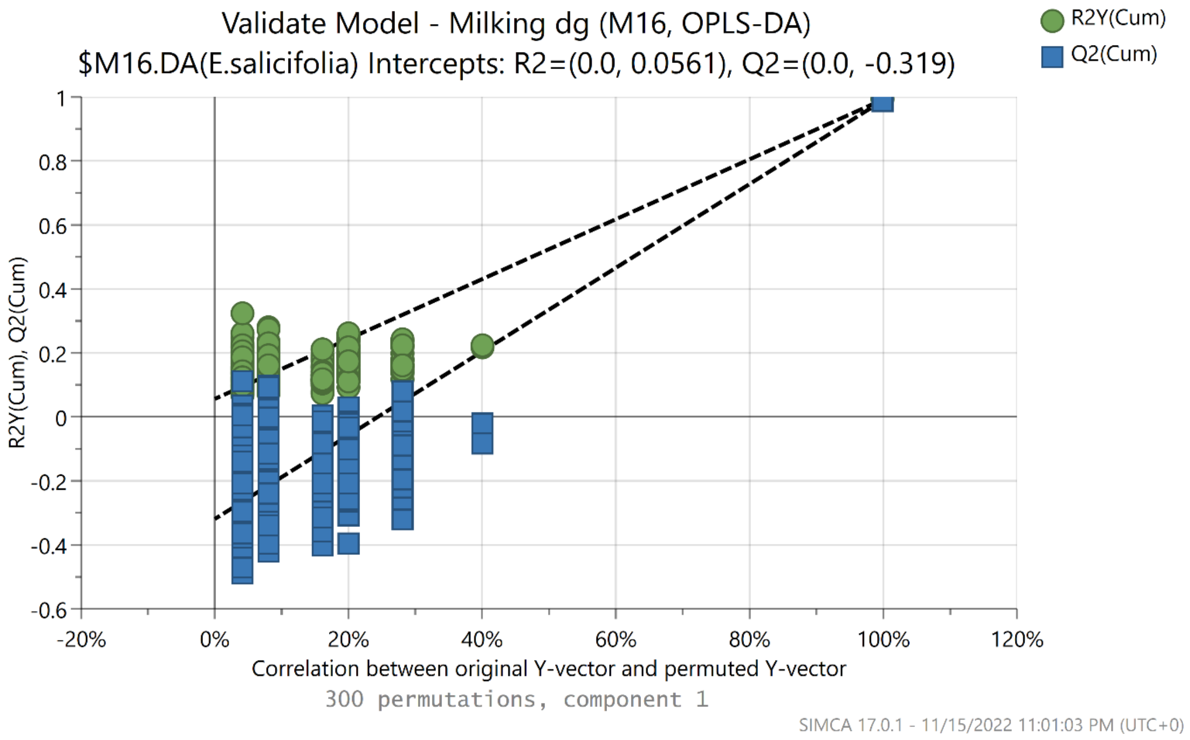

Figure S10: Permutation test of OPLS-DA model of *E. cyparissias vs*. remaining *Euphorbia* species.

Table S7. PCA and OPLS-DA model parameters of tested *Euphorbia* species.

Models									
**Number**	**Model**	**Type**	**A**	**N**	**R2X(cum)**	**R2Y(cum)**	**Q2(cum)**	**Date**	**Title**
2	M2	PCA-X	17	60	0.994		0.975	10/21/2022	
11	M11	OPLS-DA	1+3+0	60	0.609	0.993	0.988	10/23/2022	*E. segueriana vs*. remaining *Euphorbia*
12	M12	OPLS-DA	1+3+0	60	0.635	0.994	0.992	10/23/2022	*E. salicifolia vs*. remaining *Euphorbia*
13	M13	OPLS-DA	1+3+0	60	0.671	0.986	0.981	10/23/2022	*E. amygdaloides vs*. remaining *Euphorbia*
15	M15	OPLS-DA	1+2+0	60	0.595	0.993	0.991	10/23/2022	*E. panonnica vs*. remaining *Euphorbia*
14	M14	OPLS-DA	1+3+0	60	0.675	0.986	0.979	10/23/2022	*E. cyparissias vs*. remaining *Euphorbia*
16	M16	OPLS-DA	1+1+0	60	0.442	0.992	0.989	11/13/2022	*E. maculata vs*. remaining *Euphorbia*

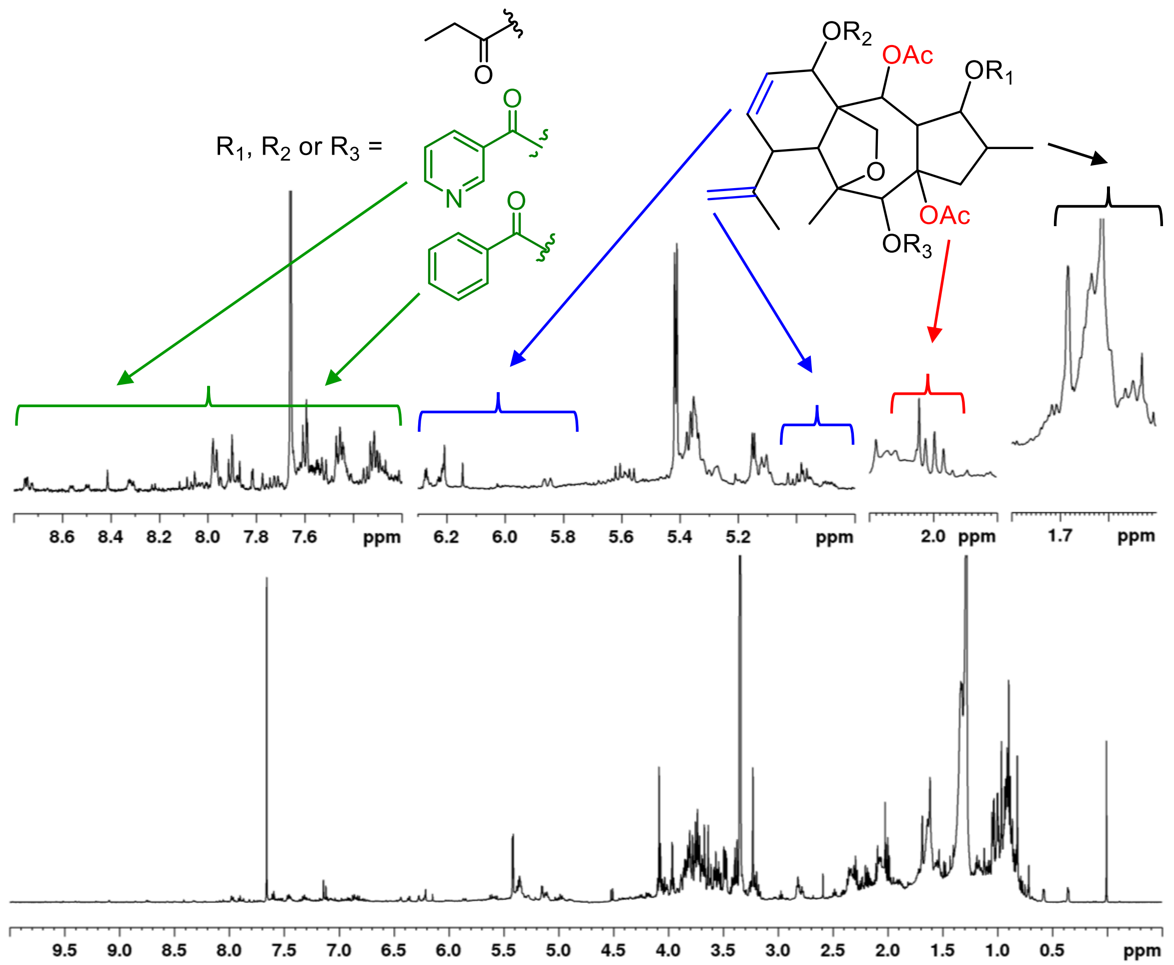

Figure S11: The ^{1}H NMR spectrum of *E. segueriana* extract obtained with MeOD:CDCl$_3$ (1:1) with assignment of characteristic resonances

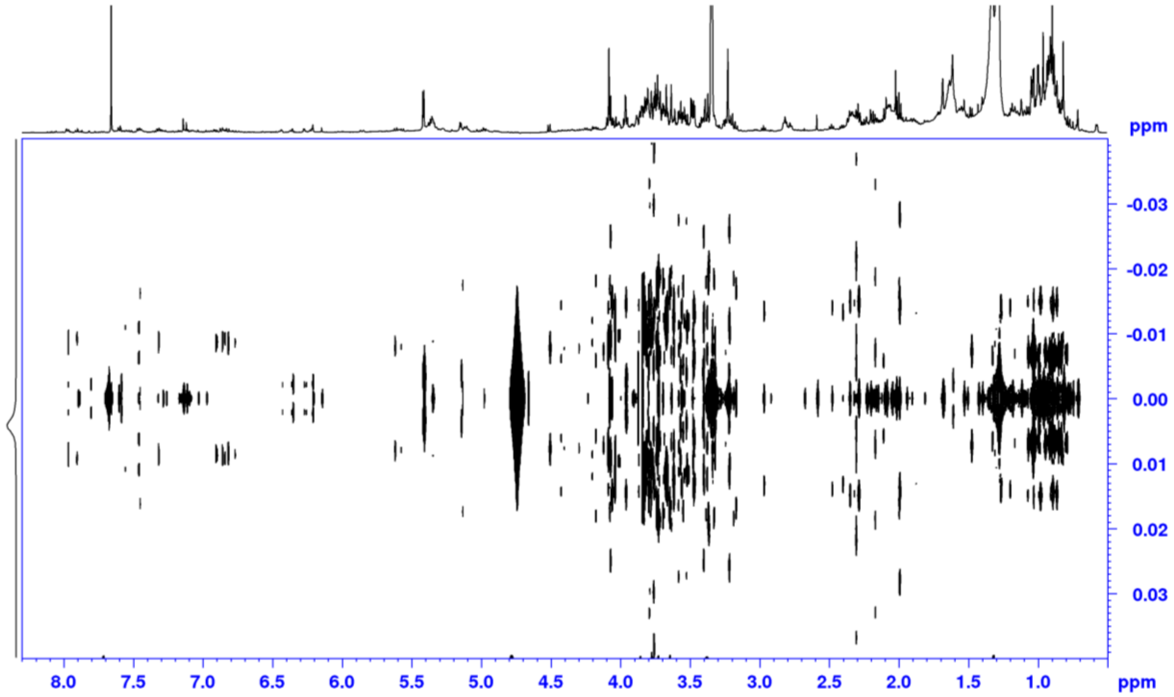

Figure S12: The H,H-*J*-resoved NMR spectrum of *E. segueriana* extract obtained with MeOD:CDCl$_3$ (1:1)

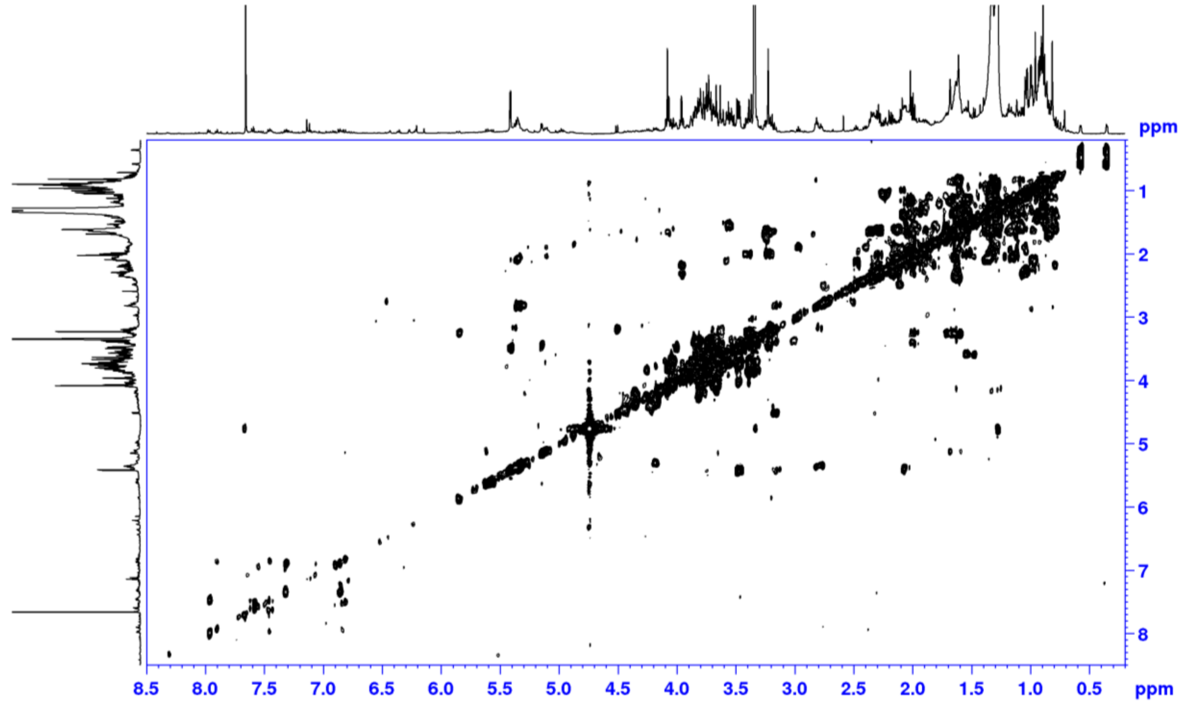

Figure S13: The COSY NMR spectrum of *E. segueriana* extract obtained with MeOD:$CDCl_3$ (1:1)

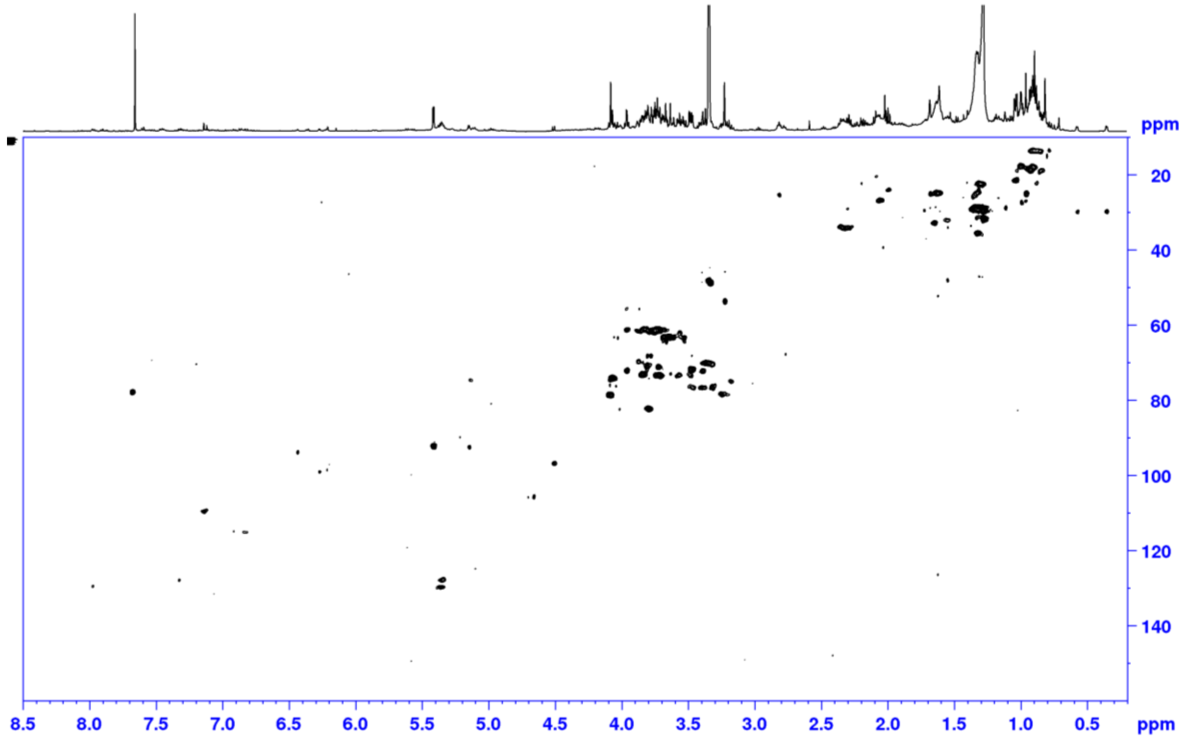

Figure S14: The HSQC NMR spectrum of *E. segueriana* extract obtained with MeOD:$CDCl_3$ (1:1)

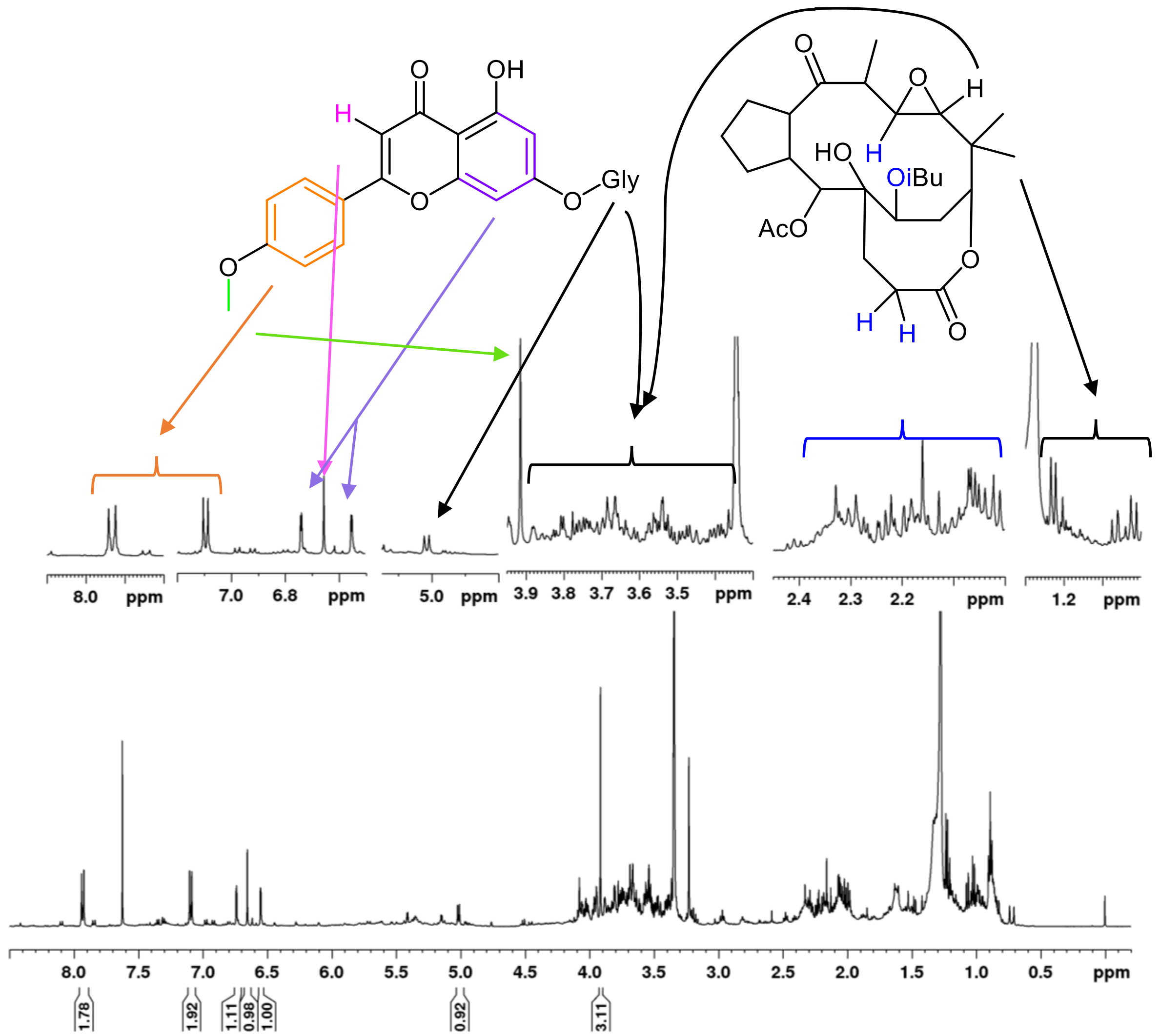

Figure S15: The ^{1}H NMR spectrum of *E. salicifolia* extract extract obtained with MeOD:$CDCl_3$ (1:1) with assignment of characteristic resonances

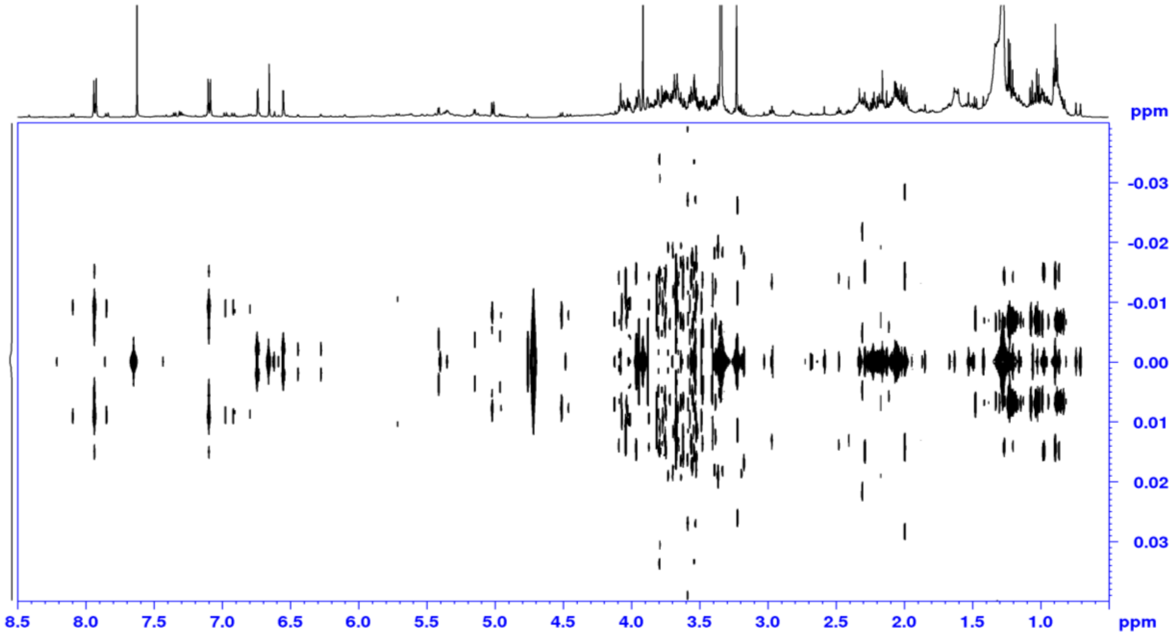

Figure S16: The H,H-*J*-resoved NMR spectrum of *E. salicifolia* extract obtained with MeOD:$CDCl_3$ (1:1)

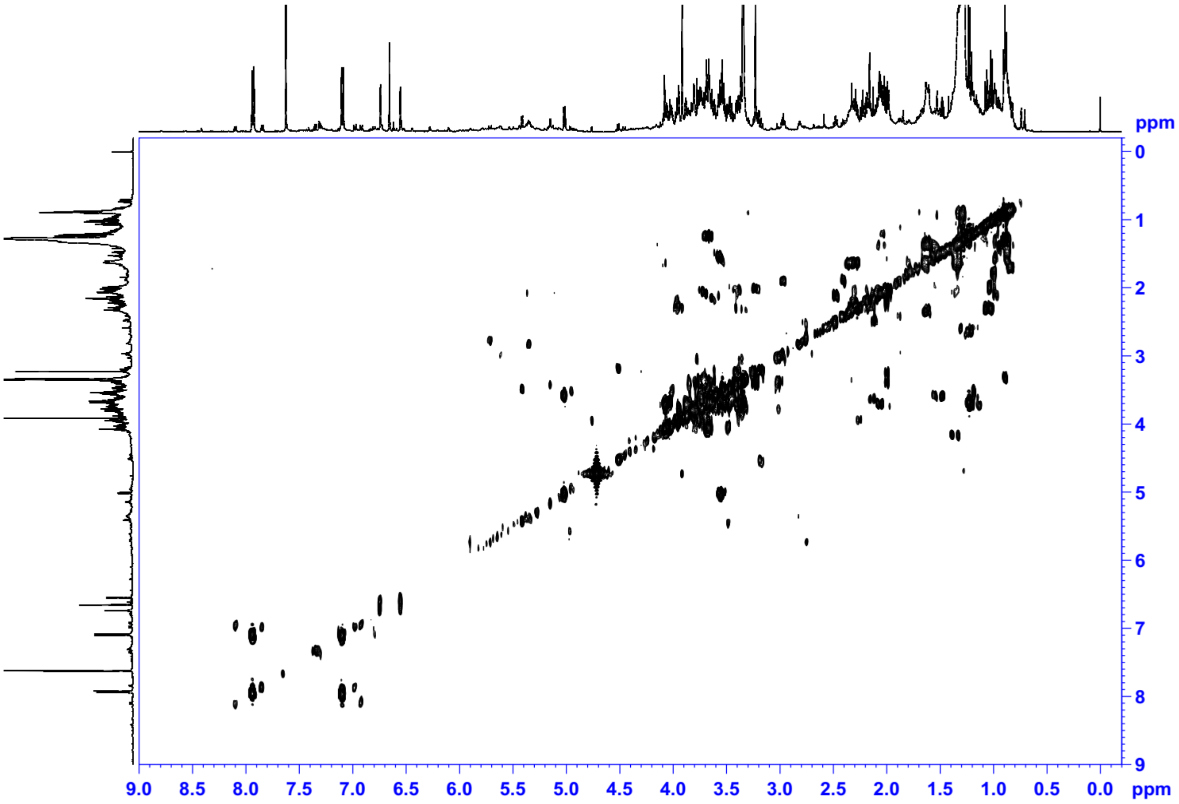

Figure S17: The COSY NMR spectrum of *E. salicifolia* extract obtained with MeOD:$CDCl_3$ (1:1)

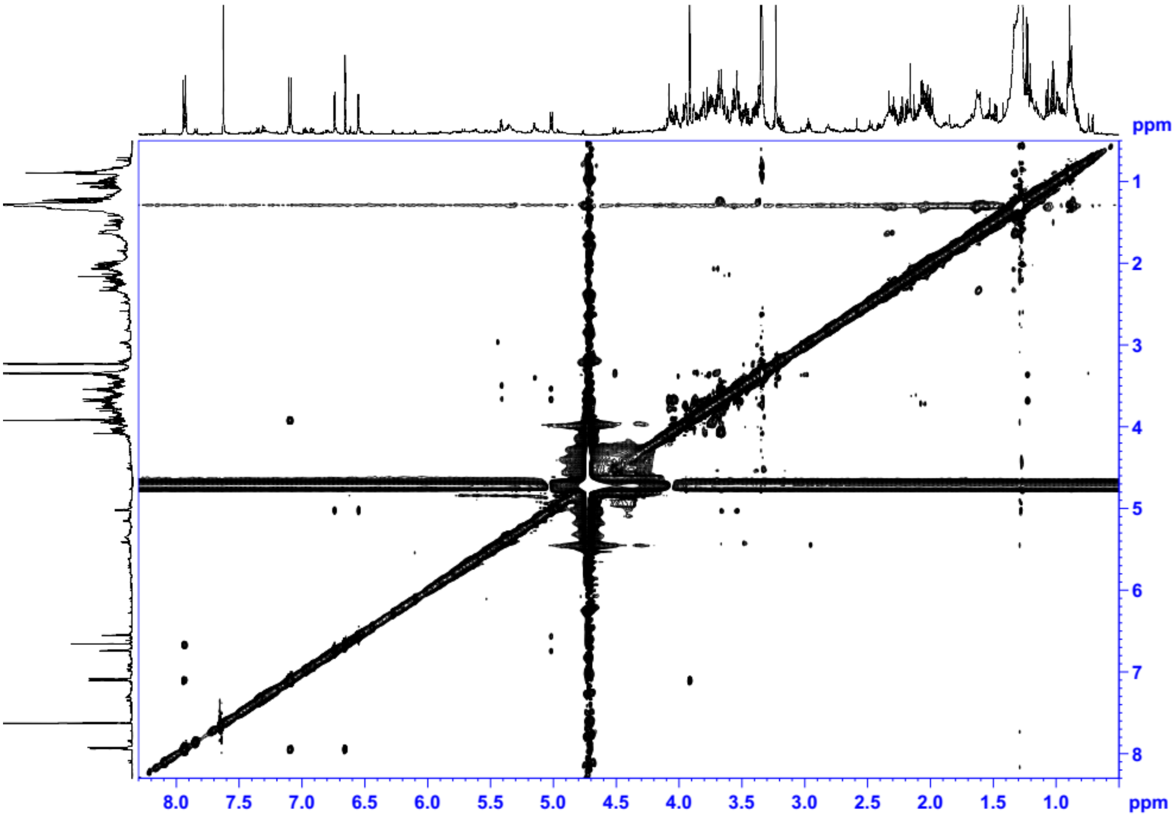

Figure S18: The NOESY NMR spectrum of *E. salicifolia* extract obtained with MeOD:$CDCl_3$ (1:1)

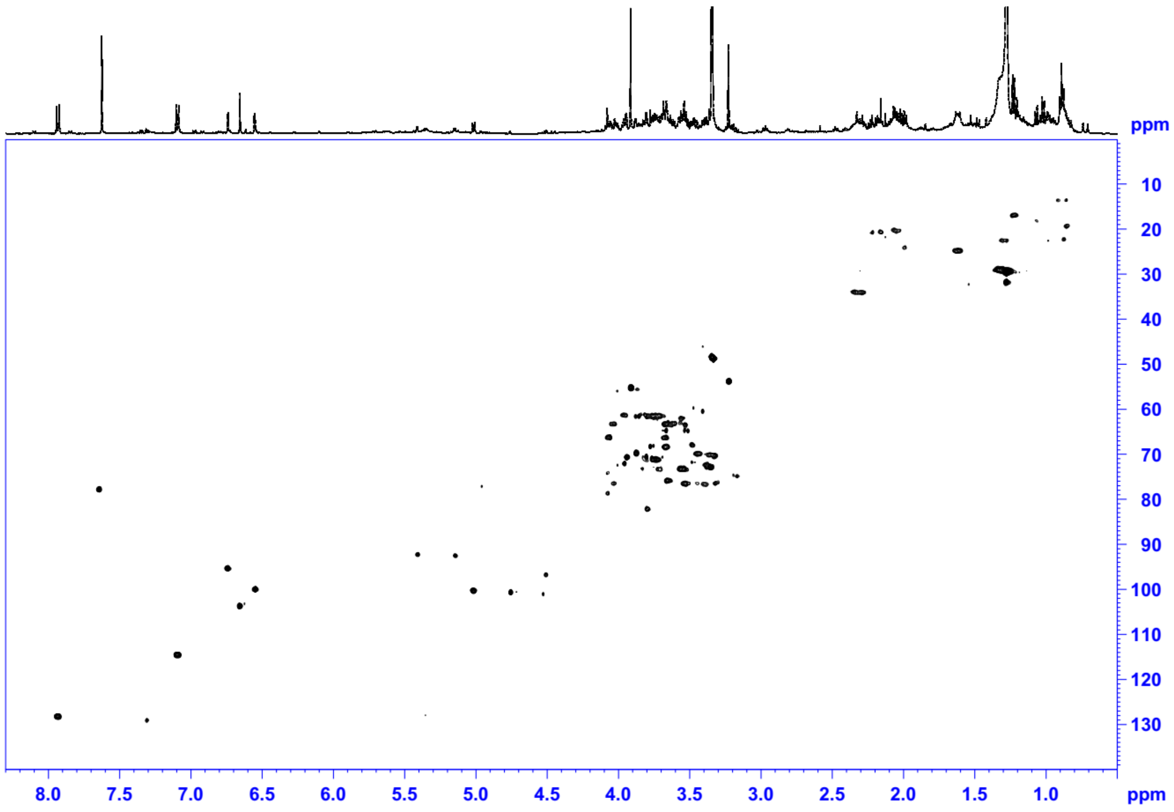

Figure S19: The HSQC NMR spectrum of *E. salicifolia* extract obtained with MeOD:$CDCl_3$ (1:1)

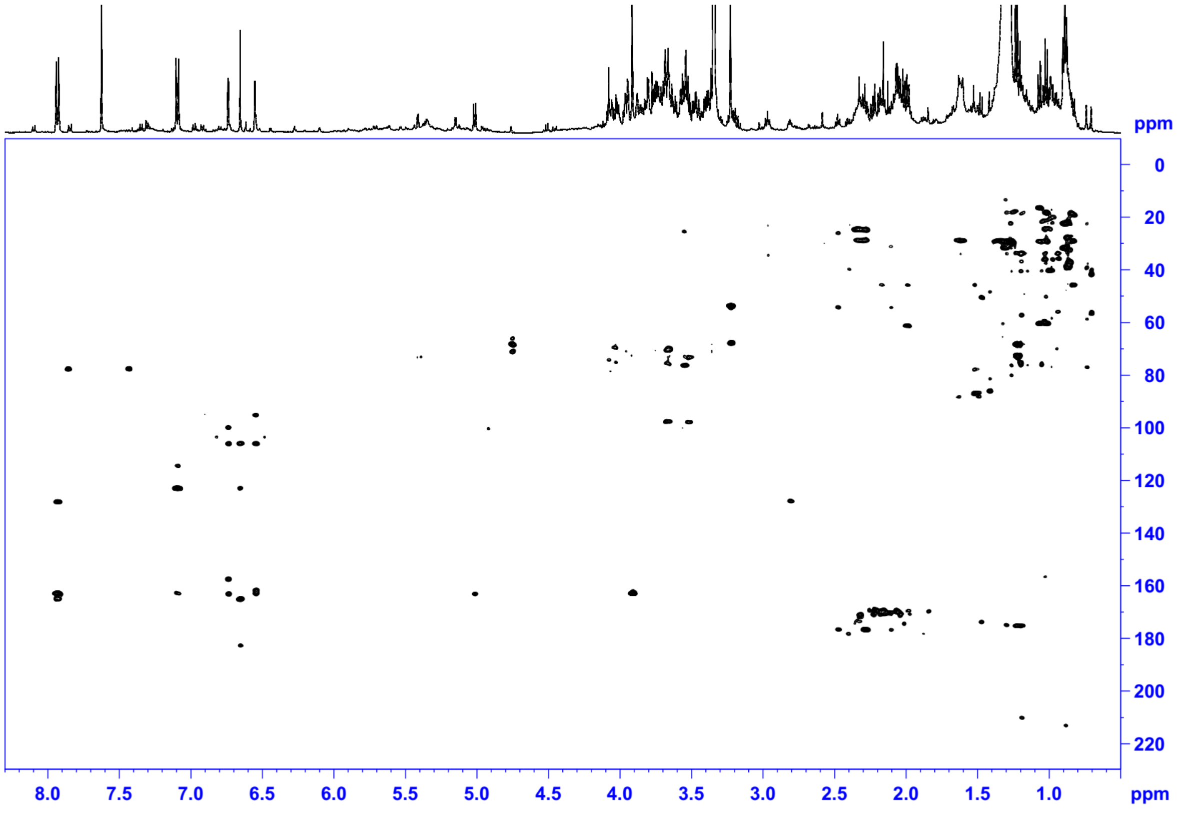

Figure S20: The HMBC NMR spectrum of *E. salicifolia* extract obtained with MeOD:$CDCl_3$ (1:1)

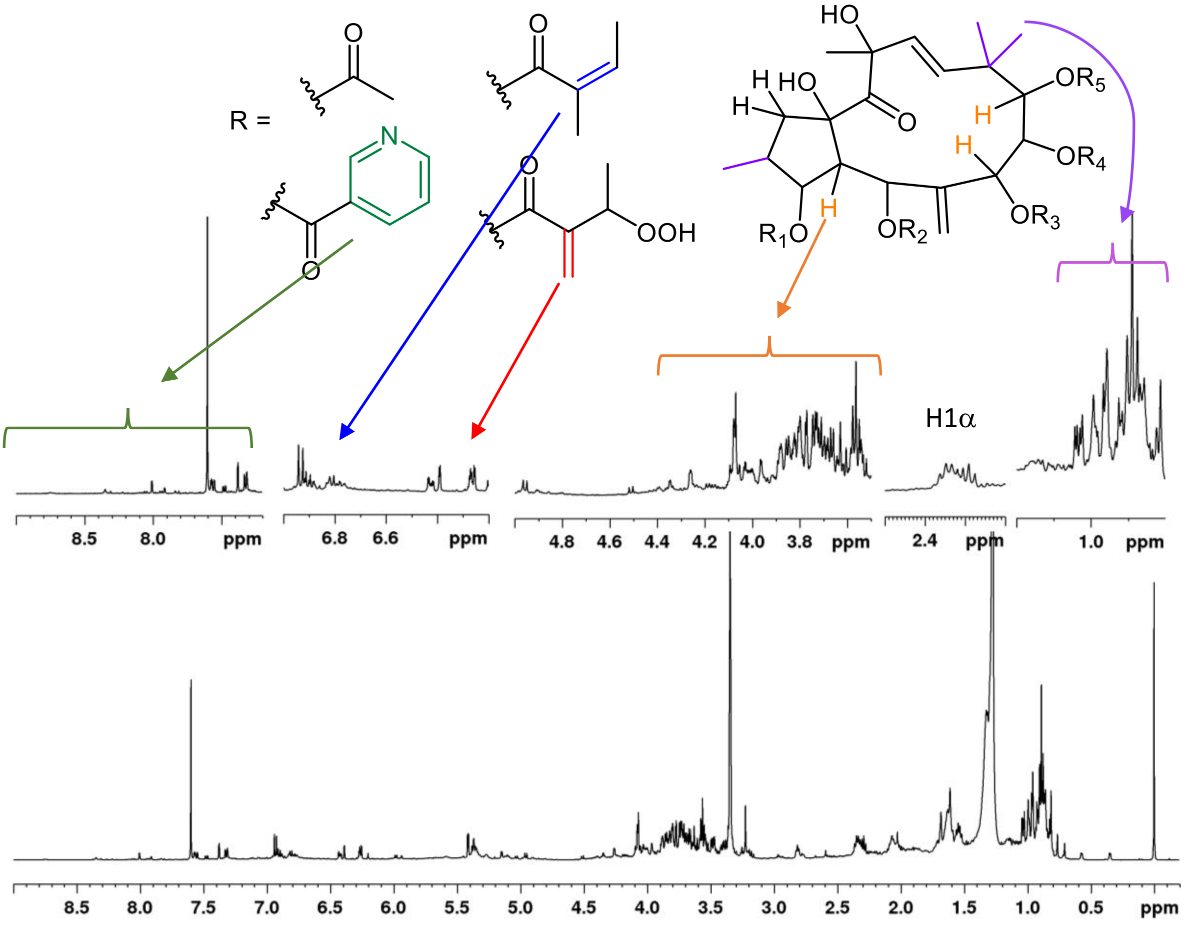

Figure S21: The ^{1}H NMR spectrum of *E. amigdaloides* extract extract obtained with MeOD:CDCl3 (1:1) with assignment of characteristic resonances

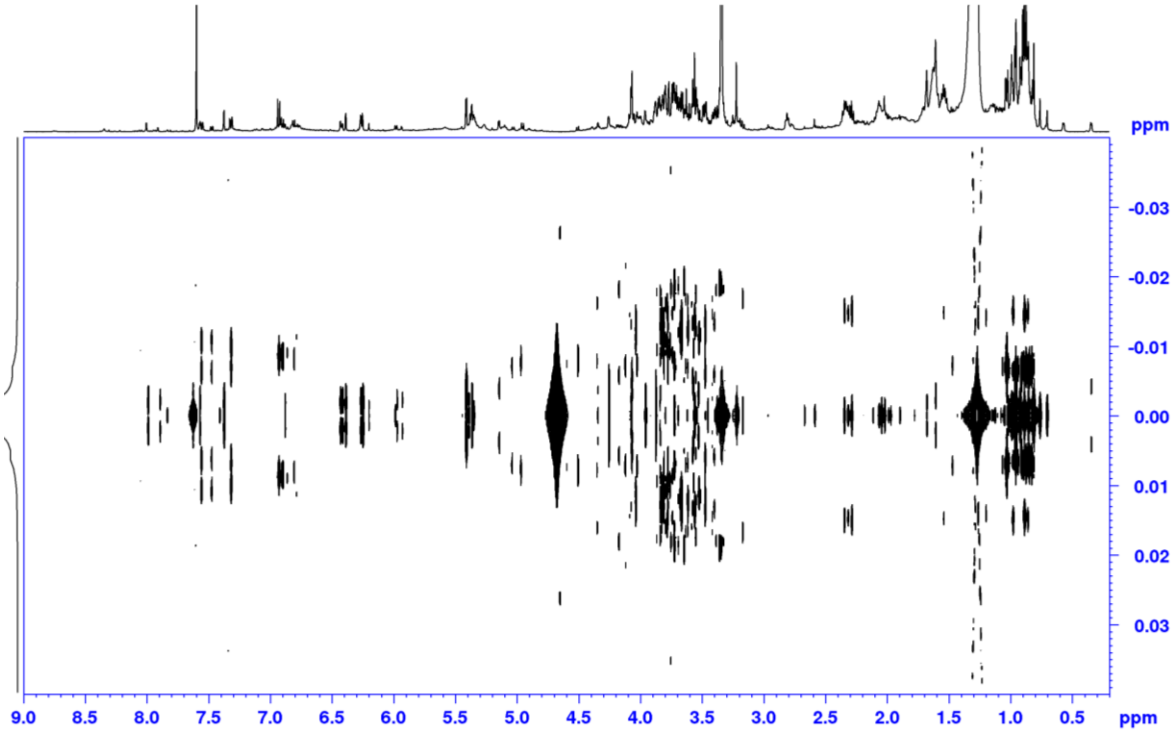

Figure S22: The H,H-*J*-resoved NMR spectrum of *E. amigdaloides* extract obtained with MeOD:CDCl3 (1:1)

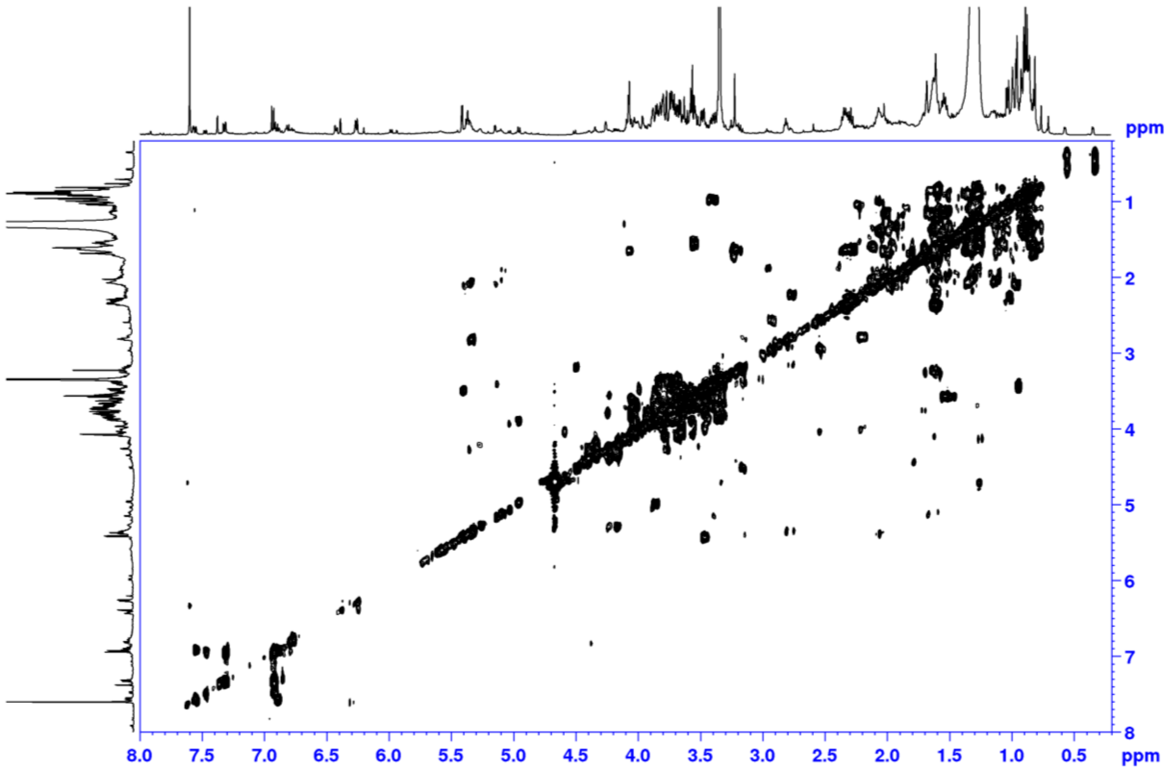

Figure S23: The COSY NMR spectrum of *E. amigdaloides* extract obtained with MeOD:$CDCl_3$ (1:1)

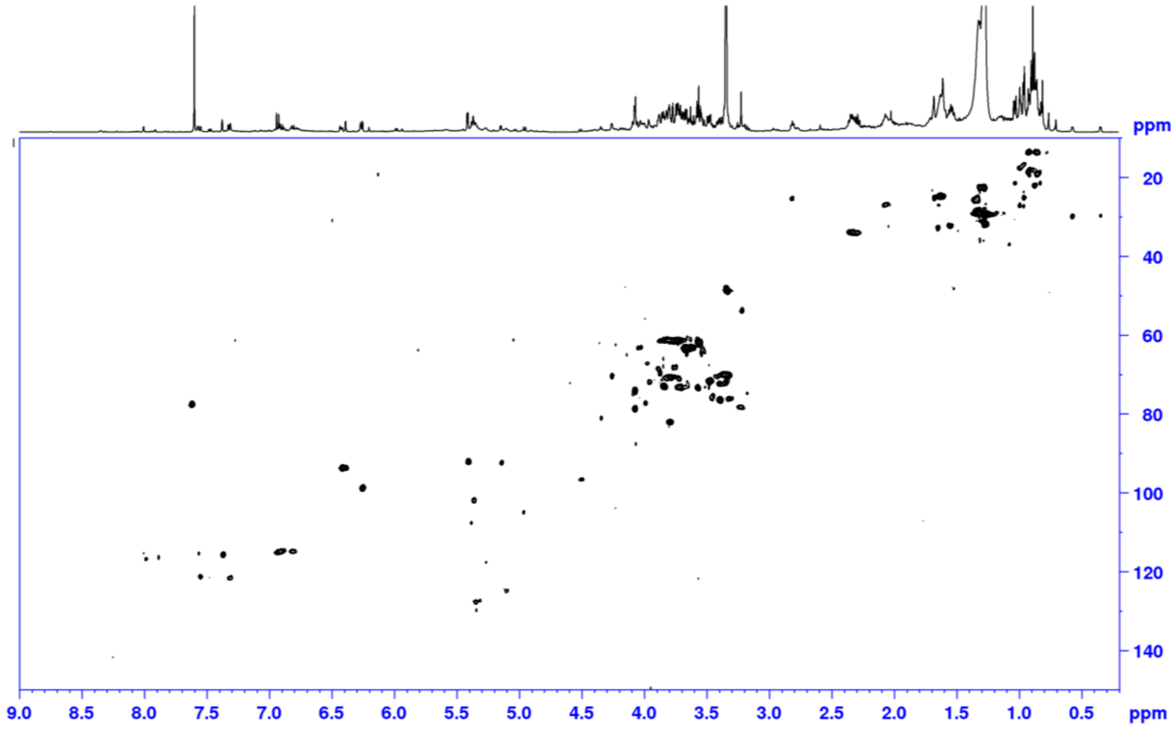

Figure S24: The HSQC NMR spectrum of *E. amigdaloides* extract obtained with MeOD:$CDCl_3$ (1:1)

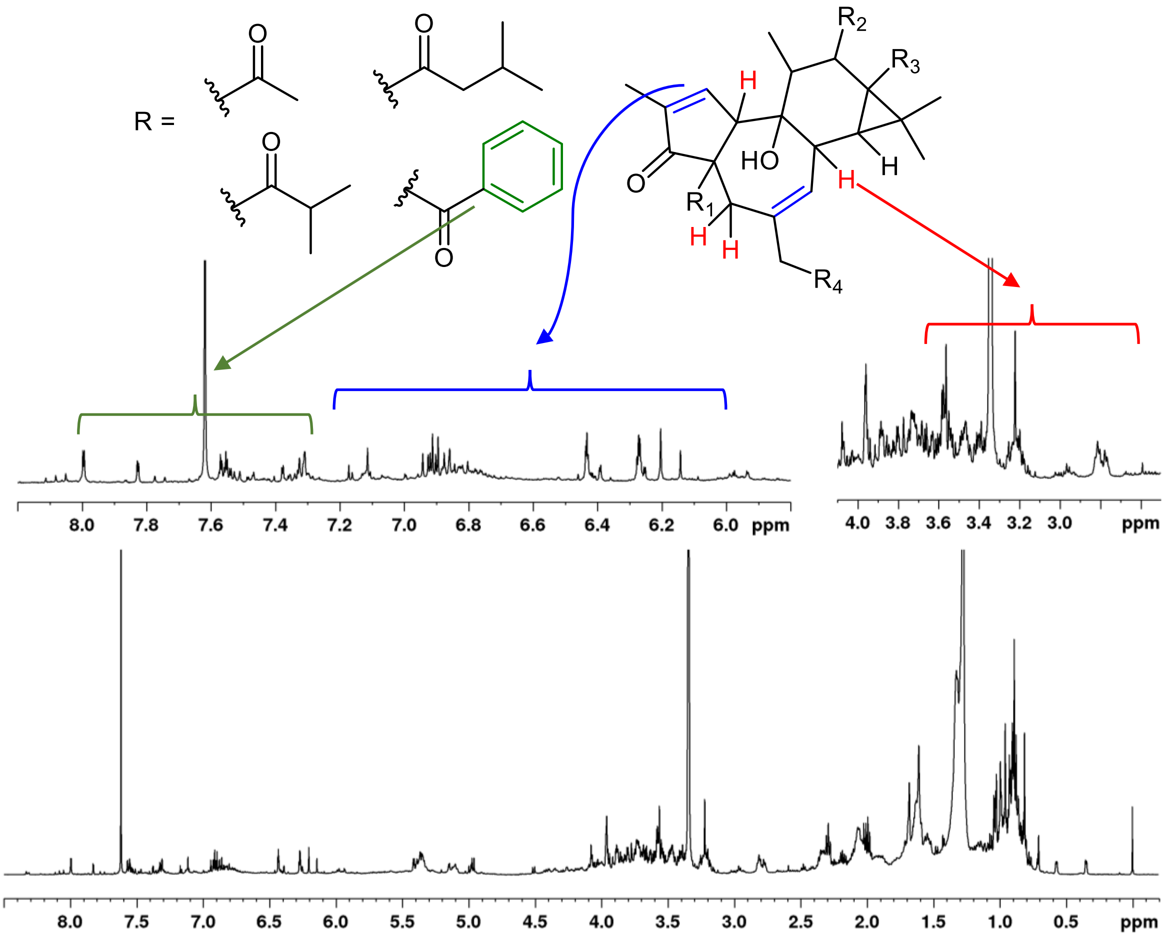

Figure S25: The ^{1}H NMR spectrum of *E. panonnica* extract extract obtained with MeOD:$CDCl_3$ (1:1) with assignment of characteristic resonances

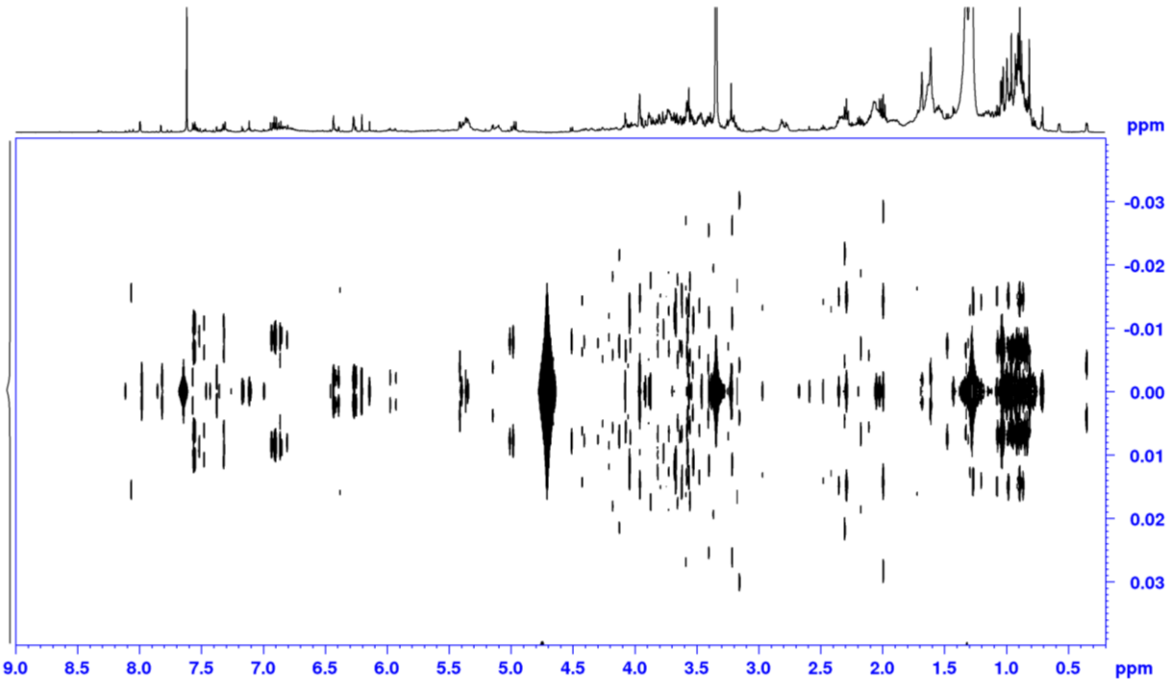

Figure S26: The H,H-*J*-resoved NMR spectrum of *E. panonnica* extract obtained with MeOD:$CDCl_3$ (1:1)

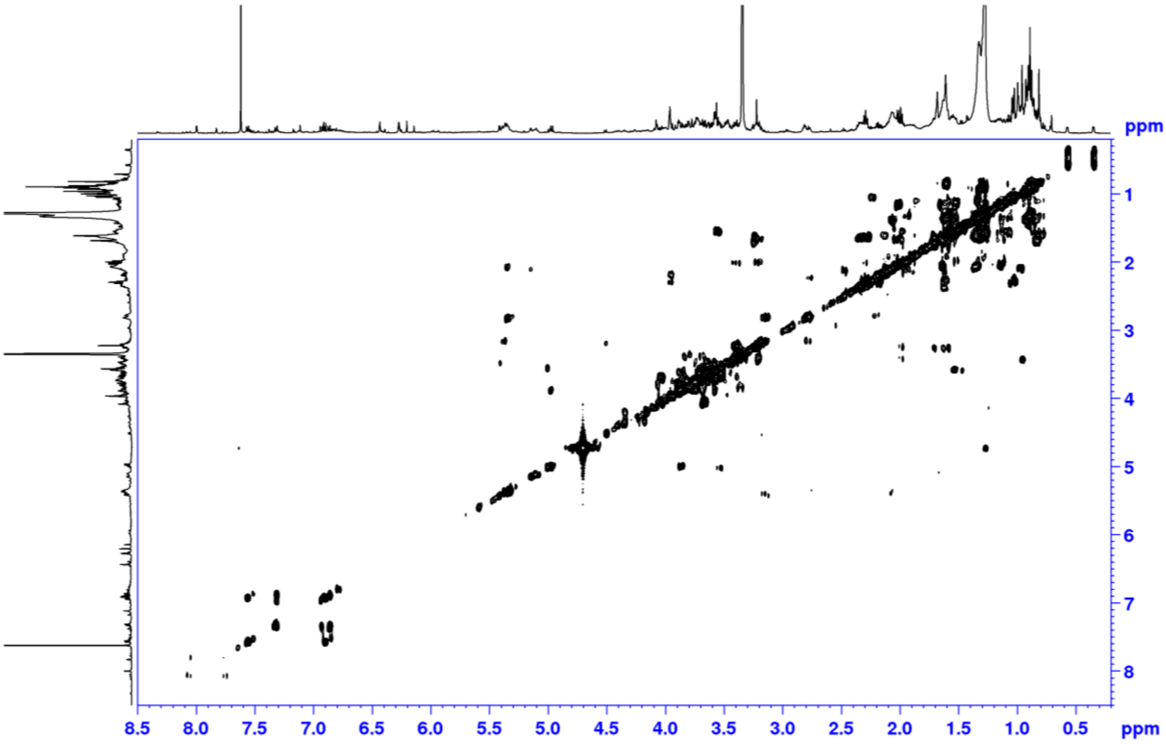

Figure S27: The COSY NMR spectrum of *E. panonnica* extract obtained with MeOD:$CDCl_3$ (1:1)

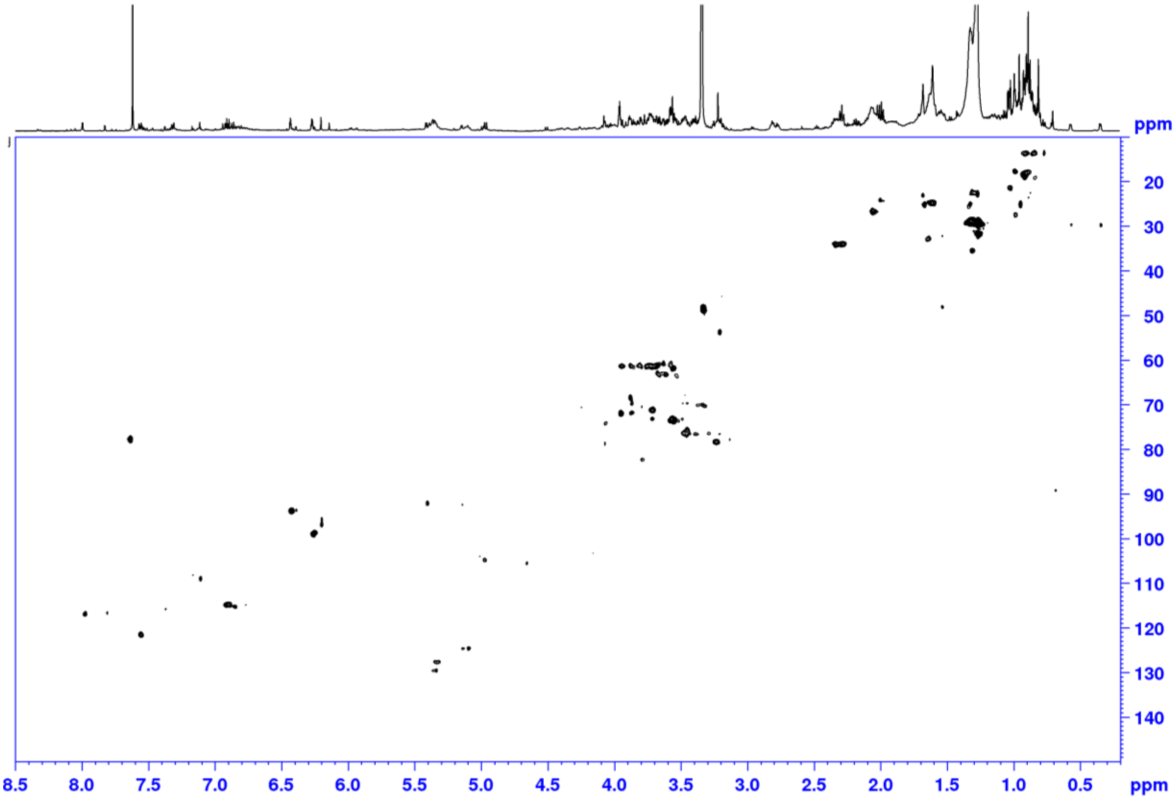

Figure S28: The HSQC NMR spectrum of *E. panonnica* extract obtained with MeOD:$CDCl_3$ (1:1)

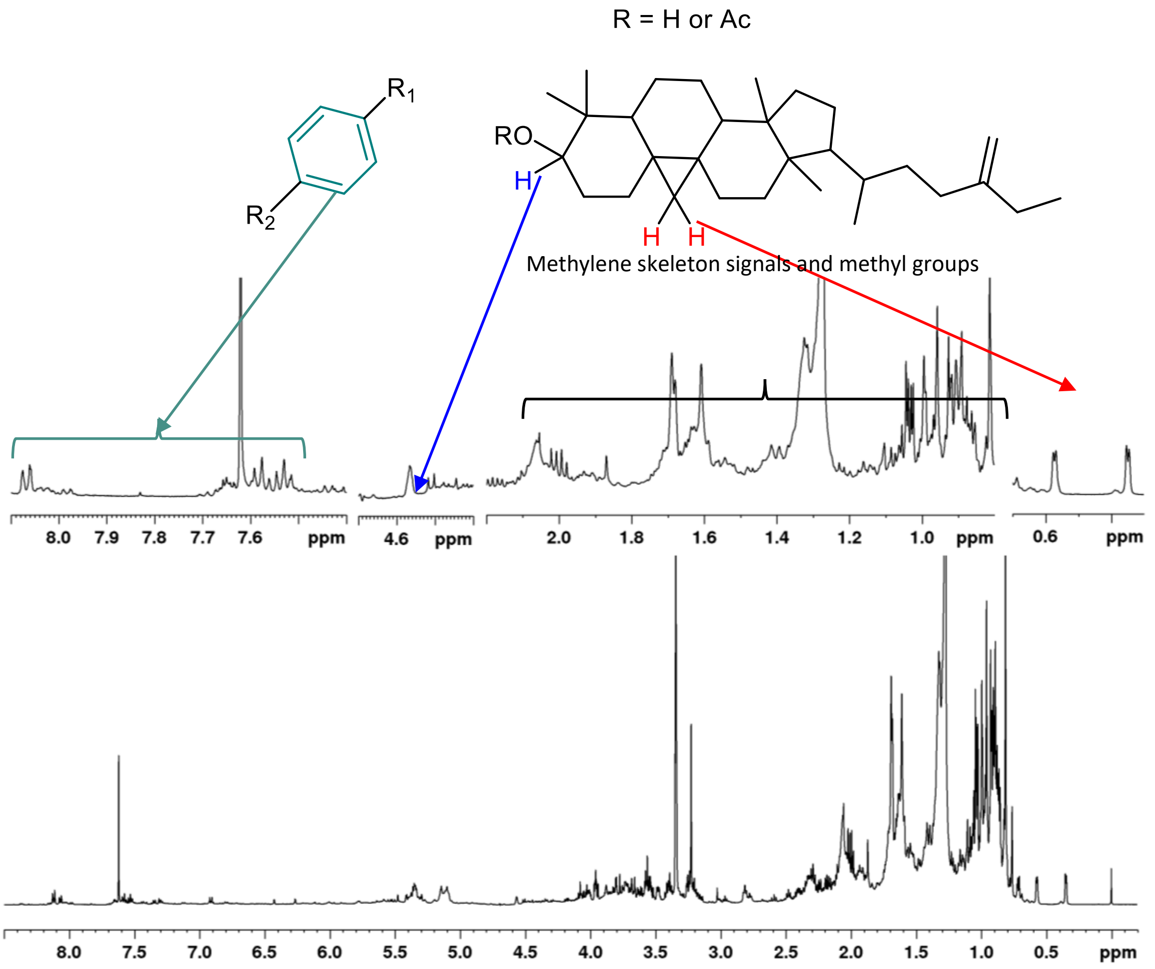

Figure S29: The ^{1}H NMR spectrum of *E. cyparisisas* extract extract obtained with MeOD:CDCl3 (1:1) with assignment of characteristic resonances

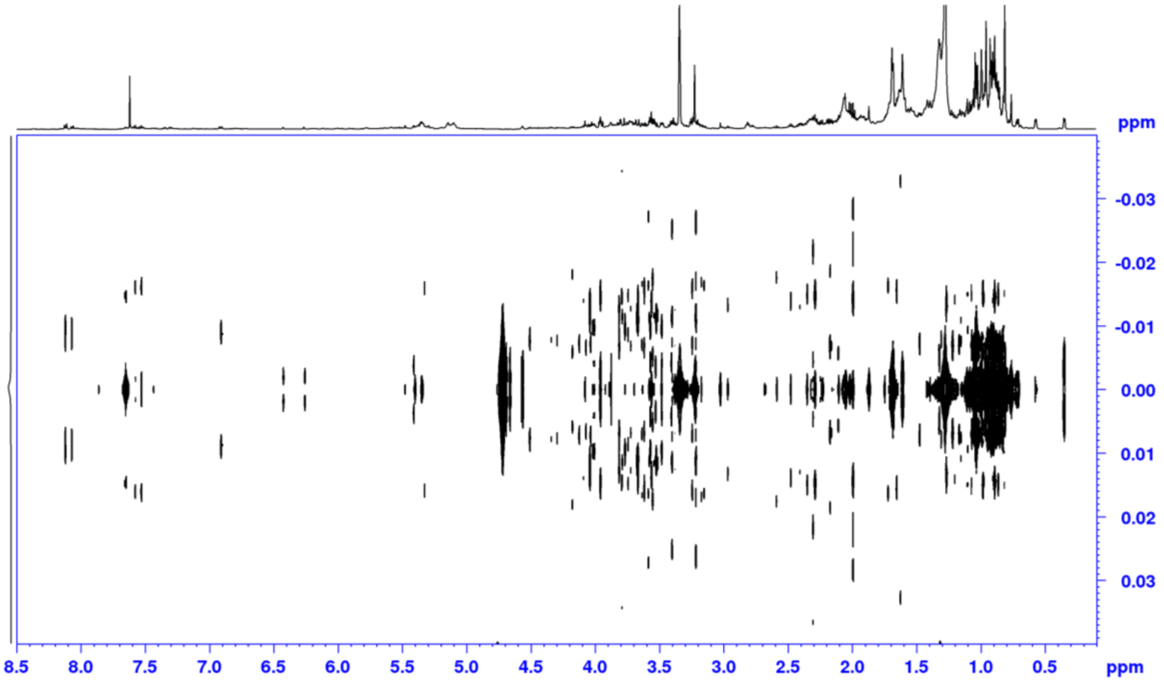

Figure S30: The H,H-*J*-resoved NMR spectrum of *E. cyparisisas* extract obtained with MeOD:CDCl3 (1:1)

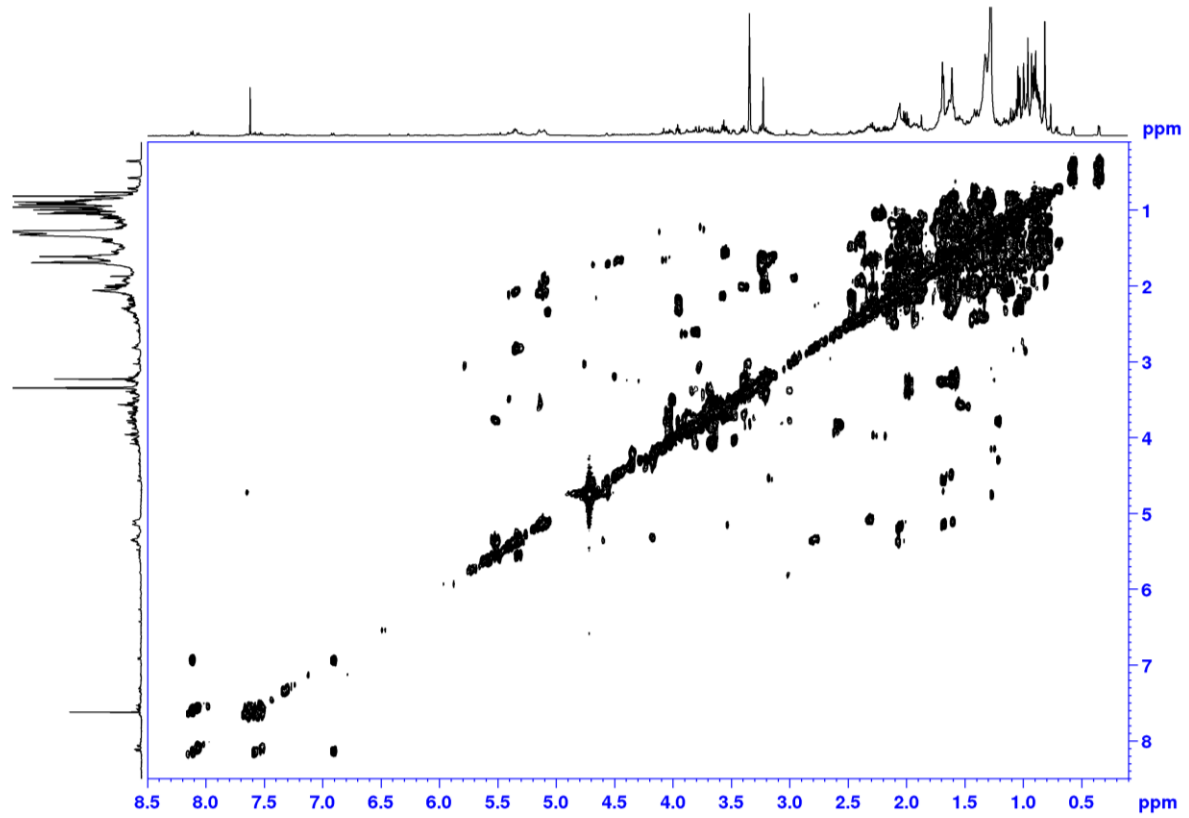

Figure S31: The COSY NMR spectrum of *E. cyparisisas* extract obtained with MeOD:$CDCl_3$ (1:1)

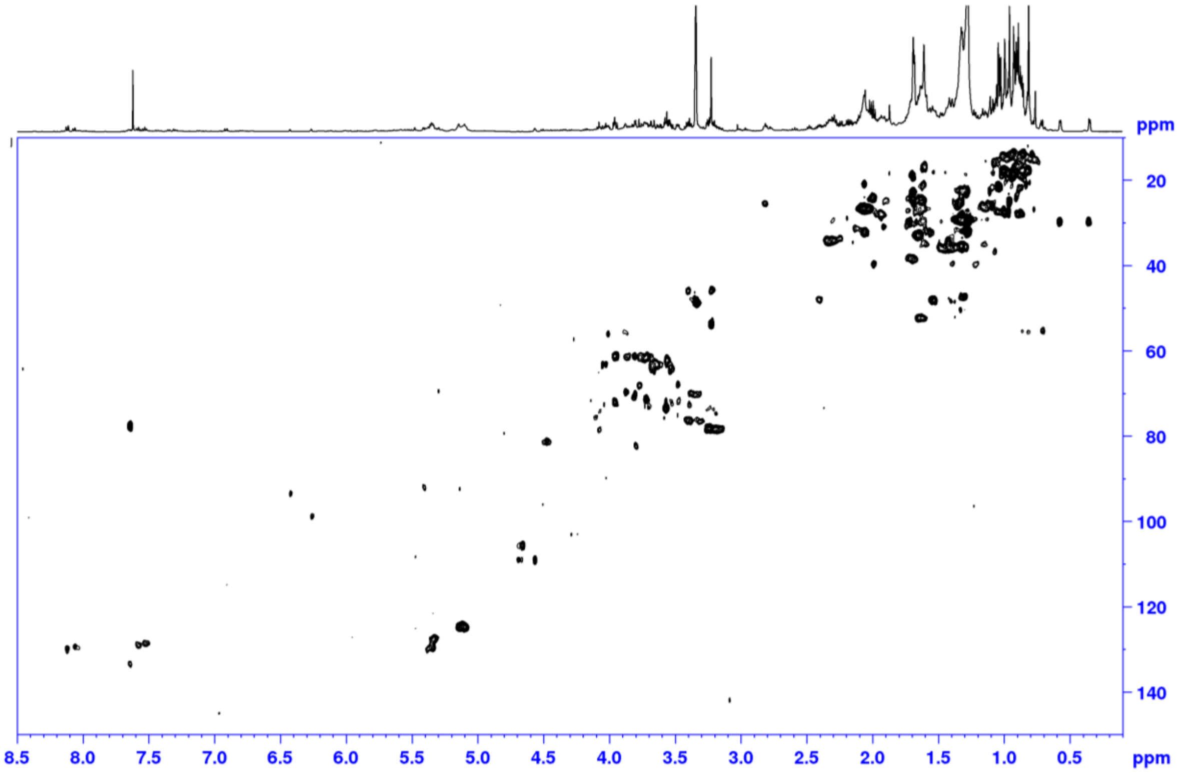

Figure S32: The HSQC NMR spectrum of *E. cyparisisas* extract obtained with MeOD:$CDCl_3$ (1:1)

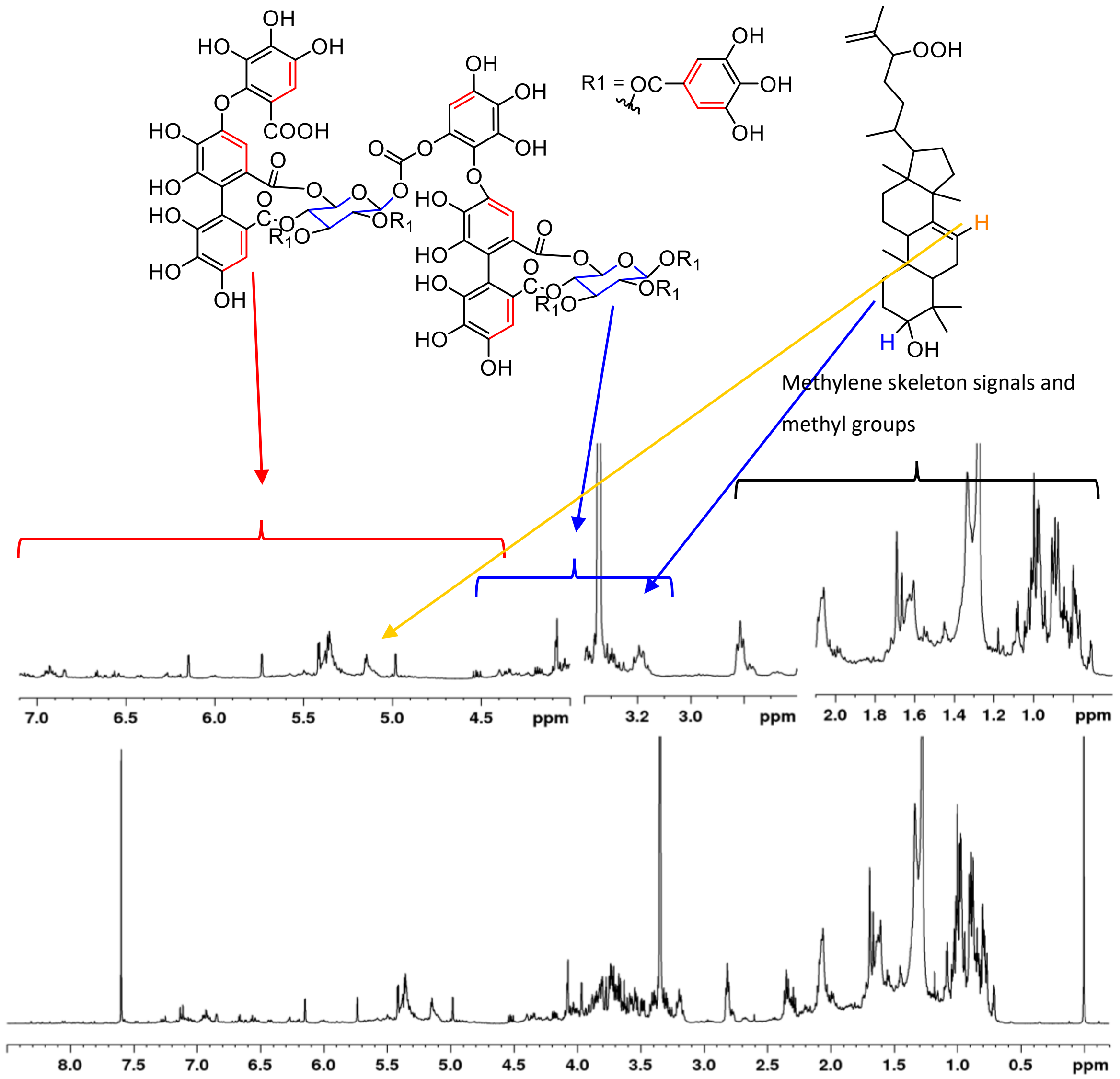

Figure S33: The ^{1}H NMR spectrum of *E. maculata* extract extract obtained with MeOD:$CDCl_3$ (1:1) with assignment of characteristic resonances

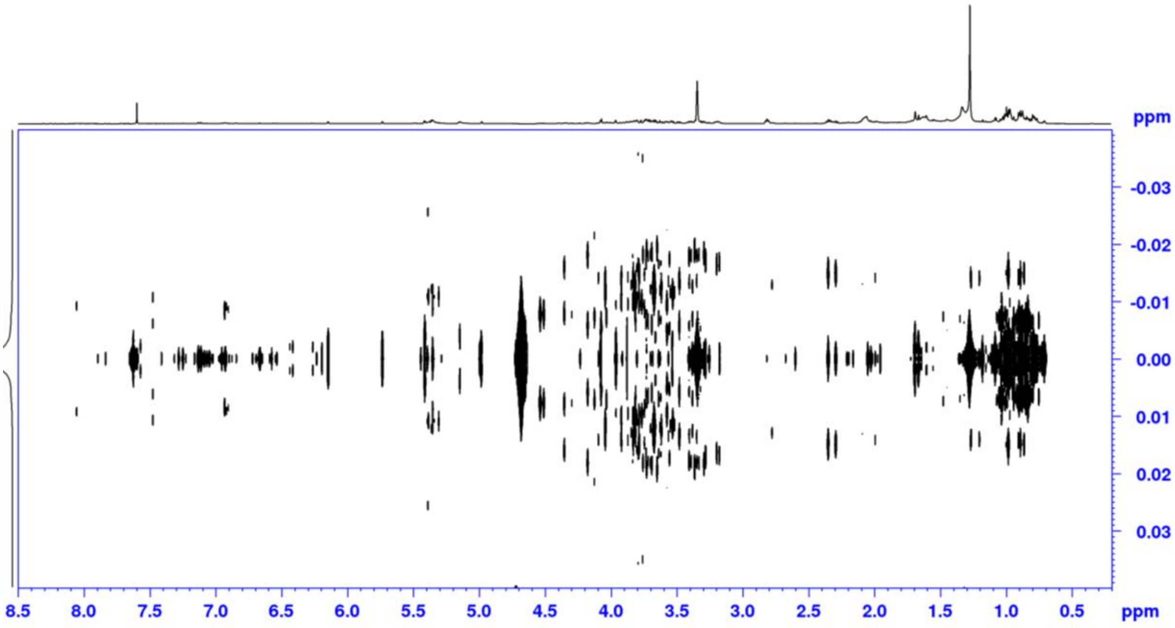

Figure S34: The H,H-*J*-resoved NMR spectrum of *E. maculata* extract obtained with MeOD:$CDCl_3$ (1:1)

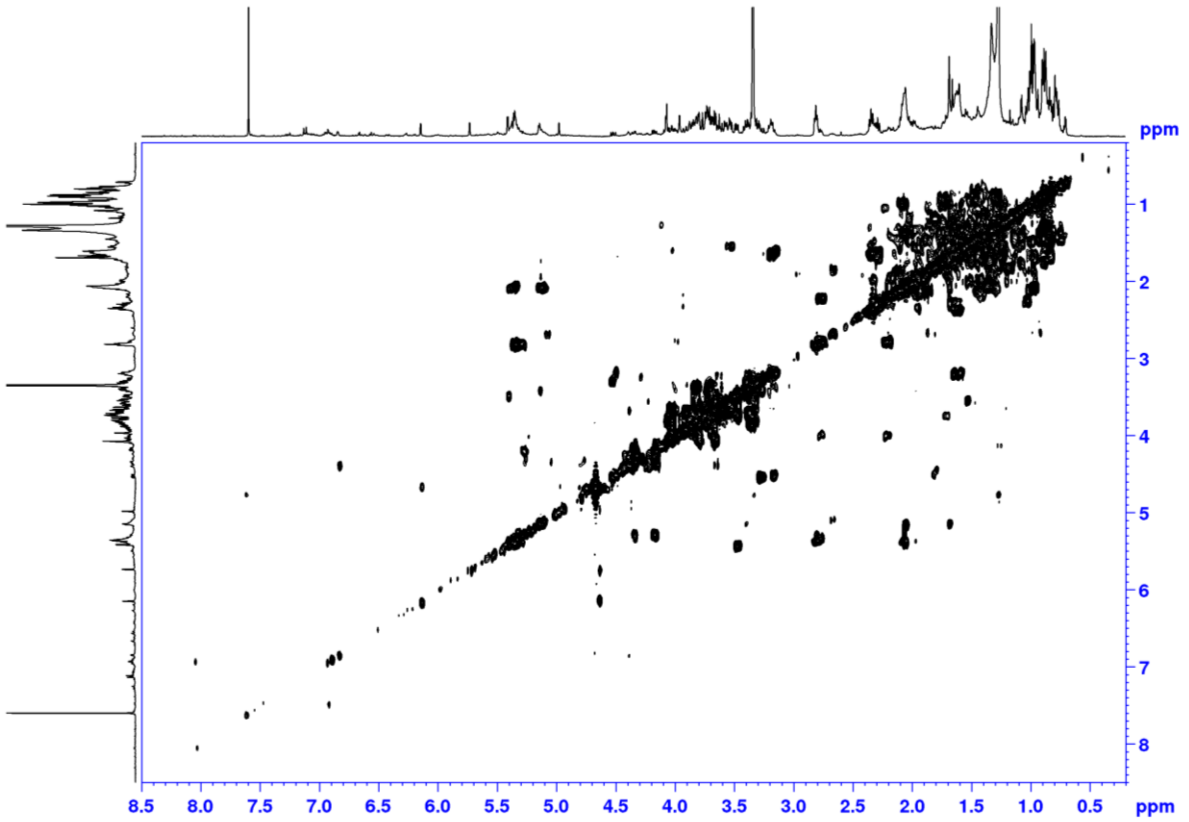

Figure S35: The COSY NMR spectrum of *E. maculata* extract obtained with MeOD:$CDCl_3$ (1:1)

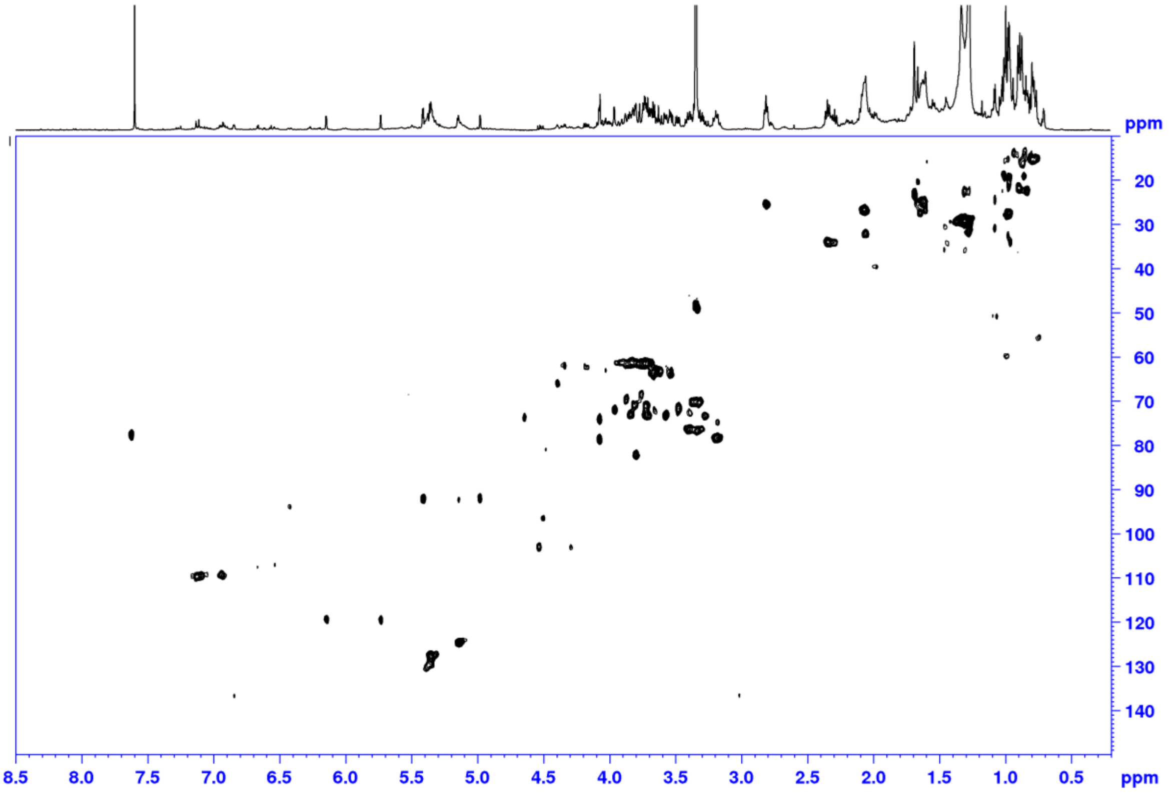

Figure S36: The HSQC NMR spectrum of *E. maculata* extract obtained with MeOD:$CDCl_3$ (1:1)
